# Neural correlates of emotion processing and regulation dissociate frontal and temporal lobe epilepsy

**DOI:** 10.1093/braincomms/fcag270

**Published:** 2026-07-28

**Authors:** Anissa Benzait, Valentina Krenz, Martin Wegrzyn, Anna Doll, Friedrich G Woermann, Kirsten Labudda, Christian G Bien, Johanna Kissler

**Affiliations:** Department of Psychology, Bielefeld University, Bielefeld 33615, Germany; Department of Epileptology, Krankenhaus Mara, Bethel Epilepsy Center, Medical School OWL, Bielefeld University, Bielefeld 33617, Germany; Department of Psychology and Neuroscience, Boston College, Chestnut Hill, MA 02467, USA; Department of Psychology, Bielefeld University, Bielefeld 33615, Germany; Department of Psychology, Bielefeld University, Bielefeld 33615, Germany; Department of Epileptology, Krankenhaus Mara, Bethel Epilepsy Center, Medical School OWL, Bielefeld University, Bielefeld 33617, Germany; Department of Magnetic Resonance Imaging, Society for Epilepsy Research, Bielefeld 33617, Germany; Department of Psychology, Bielefeld University, Bielefeld 33615, Germany; Department of Epileptology, Krankenhaus Mara, Bethel Epilepsy Center, Medical School OWL, Bielefeld University, Bielefeld 33617, Germany; Department of Epileptology, Krankenhaus Mara, Bethel Epilepsy Center, Medical School OWL, Bielefeld University, Bielefeld 33617, Germany; Department of Psychology, Bielefeld University, Bielefeld 33615, Germany; Center of Excellence Cognitive Interaction Technology (CITEC), Bielefeld University, Bielefeld 33615, Germany

**Keywords:** reappraisal, fMRI, DMN, FPCN, amygdala

## Abstract

When confronted with negative events, regulating emotions is essential for self-management and mental health. Temporal lobe epilepsy (TLE) patients, particularly right TLE (rTLE) patients, show altered responses towards emotional stimuli. However, neural correlates of emotion regulation and its interplay with stimulus-based emotion processing have not been studied in TLE so far. Therefore, we compared functional MRI activity during up- and downregulation of emotions towards negative scenes among 21 left TLE and 18 rTLE patients, 18 frontal lobe epilepsy (FLE) patients and 17 healthy controls. To assess stimulus-based processing, participants were asked to permit their feelings towards negative or neutral pictures. Voxel-based analyses were complemented by network-based analyses, as epilepsy is a network disorder. During stimulus-based emotion processing, rTLE patients displayed less activation compared to all other groups. Regions of reduced activity were mostly part of the frontoparietal control network and the default mode network (both *P*_FDR_ < 0.05) and included the frontal pole, the superior frontal gyrus, parietal regions and the cerebellum (all *P*_FWE_ < 0.05). Activation patterns during emotion regulation were highly similar between controls and left TLE and rTLE patients. Direct group comparisons revealed that during upregulation of emotions, controls and left TLE patients displayed higher activity than FLE patients in areas of the left default mode network (all *P*_FWE_ < 0.05). Our results dovetail with previous findings of diminished activity towards aversive stimuli in rTLE and show that in the same patients, neural underpinnings of emotion regulation are intact. In contrast, FLE patients displayed the opposite pattern with a largely intact response to aversive stimuli but disrupted neural underpinnings of emotion regulation, pointing to a double dissociation between rTLE and FLE.

## Teaser

Benzait *et al*. report that neural correlates of stimulus-based emotion processing are disrupted in right temporal lobe epilepsy, whereas activations during emotion regulation are preserved. In frontal lobe epilepsy, the opposite pattern was found. These results point to a double dissociation between right temporal lobe epilepsy and frontal lobe epilepsy.

## Graphical Abstract

**Graphical Abstract:**
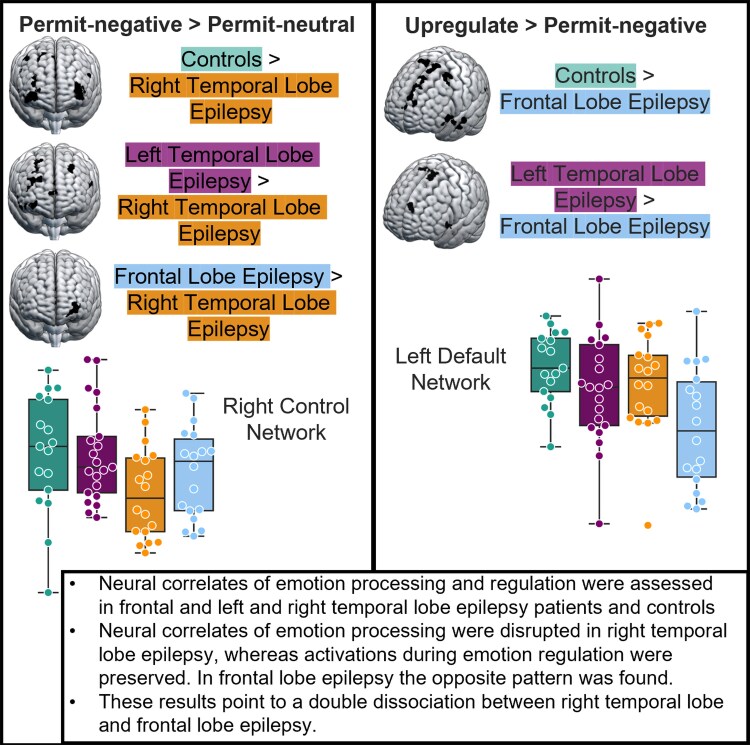

## Introduction

To date, little is known about neural networks underlying emotion regulation in epilepsy. Nevertheless, this is an issue of high clinical relevance as deficits in emotion regulation are related to psychological disorders^1^ and as living with epilepsy is an everyday emotional burden. Reappraisal is a cognitive emotion regulation strategy that is core to cognitive behavioural therapeutic interventions^2^ and has been the subject of extensive neuroimaging research.^3^ Studies using reappraisal have consistently shown that the amygdala is a major target of emotion regulation efforts.^4,5^ Amygdala responsiveness to emotional stimuli is reduced in temporal lobe epilepsy (TLE) patients compared to healthy controls (HC).^6-9^ TLE is the most common type of focal epilepsy.^10^ Focal epileptic seizures originate within networks limited to one hemisphere.^11^ In particular, right TLE (rTLE) or right temporal lobe resections have been shown to diminish stimulus-based emotion processing^8,12-14^ (but see Bonelli *et al*.^7^ for contradictory evidence), potentially mirroring a specific role of the right amygdala in bottom-up-driven relevance signalling.^15^

Very little research has addressed emotion regulation in TLE. One study found that TLE patients reported more emotion regulation difficulties than controls.^16^ In line with evidence that emotion regulation relies on cognitive brain networks that strongly depend on frontal lobe integrity,^3,17^ we recently demonstrated disrupted brain activity in frontal lobe epilepsy (FLE) patients during emotion regulation.^18^ However, TLE is less related to deficits in executive functions than FLE.^19,20^ Thus, in TLE, activation patterns during reappraisal might be more similar to those of HC. Yet, disrupted reactivity towards emotional stimuli in TLE might interact with regulation efforts and impact top-down control. Moreover, reappraisal relies on semantic and language networks that are particularly affected by left TLE (lTLE).^21,22^ As cumulative evidence points to a right hemispheric dominance in emotion processing, attention and inhibitory control,^23^ rTLE patients may also display altered activation patterns during emotion regulation.

In the present study, haemodynamic correlates of emotion regulation and stimulus-based emotion processing were assessed in HC, lTLE, rTLE and FLE, using functional MRI (fMRI). We use the term ‘stimulus-based’ to refer to natural, unregulated responses elicited by emotional stimuli and to distinguish it from goal-directed emotion regulation. Participants were asked to upregulate or downregulate their feelings towards negative pictures using reappraisal or to permit their feelings towards negative or neutral pictures.

Previous results regarding the strong interconnectedness of the frontal and temporal lobes,^24^ the widespread propagation of epileptic activity beyond the epileptic focus and interictal alterations in large-scale resting-state network connectivity in FLE and TLE^25-27^ have raised the question of to what extent and in which ways these epilepsy syndromes are distinguishable entities.^19,28^ To examine whether our results were specific to TLE or represent more general alterations related to focal epilepsy, lTLE and rTLE patients were compared to HC and FLE patients. As epilepsy is known to affect large-scale neural networks,^26,27^ traditional voxel-based analyses were complemented by investigating task-specific recruitment of established resting-state networks. We expected that during stimulus-based emotion processing, activity in task-relevant regions would be reduced in rTLE patients compared to controls. We further assumed that during stimulus-based emotion processing, rTLE patients would also show less activity than lTLE and FLE patients, given that FLE and lTLE patients have been inconsistently found to differ from HC during emotion perception, and if at all, differences were restricted to a few circumscribed regions, including the epileptic focus region.^6,8,18,29^ Based on the above-outlined mechanisms of reappraisal, we expected that during emotion regulation, lTLE and rTLE patients would exhibit reduced task-related activity than controls, but still stronger activations than FLE patients.

## Materials and methods

### Participants

We included 21 lTLE, 18 rTLE and 18 FLE patients (11 left-sided, three right-sided, three bilateral and one with unclear focus lateralization) without any additional established neurological disorder or intellectual disability. Diagnoses of TLE and FLE were based on semiology, MRI and EEG findings (see Supplementary Table 1). Two findings had to point to the same epilepsy syndrome, while one finding was allowed to be inconclusive or even incompatible. In a few individual cases, a single convincing diagnostic result with two inconclusive or normal findings was considered sufficient. The diagnoses were critically reviewed and confirmed by the epileptological co-author of the study, a board-certified neurologist, epileptologist and electrophysiologist, CGB. Five patients had additional brain lesions that were unrelated to epilepsy aetiology: post-traumatic in the temporal (*n* = 1) or frontal regions (*n* = 1); post-ischaemic in the right nucleus caudatus (*n* = 1); and in the white matter of the fronto-insular (*n* = 1) or frontoparietal regions (*n* = 1). Patients with previous brain surgery were excluded from participation, except one lTLE patient who had undergone tumour biopsy. Additionally, participants who gave erroneous explanations of experimental conditions after completing the fMRI task (one rTLE and one FLE patient) were excluded from data analysis. One HC was excluded due to excessive head movements during scanning. Patients were recruited during an inpatient stay at the Department of Epileptology, Krankenhaus Mara, Bethel Epilepsy Center, Medical School OWL, Bielefeld University, Germany.

Seventeen healthy participants without reported psychiatric or neurological disorders were included as controls. Their data have been reported previously, along with FLE group data.^18^ Controls were paid 25 Euros for participation. All participants, including patients, provided informed consent according to the Declaration of Helsinki prior to participation and were fluent in German. This research was approved by the University of Bielefeld ethics committee (application no. 2017-006).

Detailed information on sample characteristics are reported in the Results section.

### Emotion regulation paradigms

**Figure 1:**
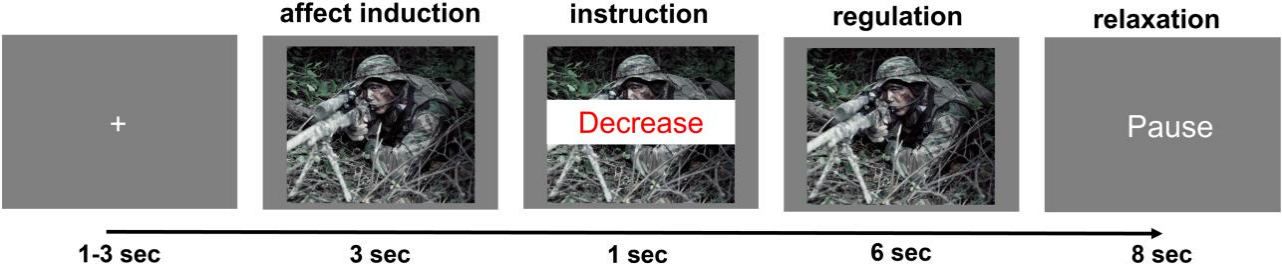
**Example of one functional magnetic resonance imaging trial in the downregulate-negative condition.** In the behavioural task, instead of a relaxation phase, participants were asked to rate the intensity of their negative feelings on a 7-point Likert scale at the end of each trial.

Either 1 or 2 days before fMRI scanning, the emotion regulation task was practiced. Participants were instructed to either up- or downregulate their emotions towards negative pictures from the International Affective Picture System^30^ using self-generated reappraisals or to permit their emotions, i.e. respond naturally, towards negative or neutral pictures. In the behavioural emotion regulation task, participants were asked to rate the intensity of their negative feelings on a 7-point Likert scale ranging from 1 = ‘very weak’ to 7 = ‘very strong’ after each trial. Figure 1 shows an example trial of the fMRI paradigm. The order of conditions was pseudo-randomized in that each instruction was presented at least twice and a maximum of three times in a row. During the behavioural task, each condition (‘permit-neutral’, ‘permit-negative’, ‘downregulate-negative’, ‘upregulate-negative’) was presented 12 times in two runs. The fMRI task was split into four runs, with each condition being presented six times within one run, resulting in 24 trials per condition. The whole fMRI task lasted about 35 min. To ensure correct application of reappraisal strategies, the task was shortly practiced again before fMRI data acquisition, and it was ensured that all participants were able to verbalize adequate reappraisals. Please see Benzait *et al*.^18^ for more details about the emotion regulation paradigm for behavioural and fMRI testing. Both paradigms were programmed and presented with the software package PsychoPy2 (version 1.84.2).^31^

### Behavioural data analysis

Behavioural data were analysed using SPSS 25 (IBM Corp., released 2017). Repeated measures analyses of variance or covariance were conducted for group comparisons and within-group analyses. Current age and age at epilepsy onset were included as covariates in analyses of covariance (ANCOVAs) comparing HC and TLE patients and FLE and TLE patients, respectively. Significance levels were set to *P* < 0.05, two-tailed. *Post hoc* comparisons were Bonferroni corrected. Greenhouse–Geisser adjustment was used to correct for violations of sphericity where necessary. Uncorrected degrees of freedom are reported for better readability.

### MRI data acquisition

Collection of MRI data was performed with a 3 T Siemens Verio Scanner at The Mara, Department of Epileptology, Bielefeld University. A 32-channel head coil was used to acquire 192 sagittal slices for high-resolution T1-weighted 3D images (0.8 mm thickness, 15.36 × 24 × 24 cm field of view, 0.75 × 0.75 mm in-plane resolution, 2.5 ms echo time, 1.9 s repetition time). A 12-channel head coil was used to acquire functional gradient-echo planar T2 images. Thirty-five axial slices (4 mm thickness, 4 mm gap, interleaved order) were acquired with 33 ms echo time, 3 s repetition time, 90° flip angle and 15.36 × 24 × 24 cm field of view. Orientation of images was parallel to the anterior–posterior commissure line. Steady-state magnetization was ensured by discarding three scans at the beginning of each run.

### MRI data preprocessing

For preprocessing of structural and functional MRI data, we used fMRIPrep 1.3.0.post2.^32^ T1-weighted images were corrected for intensity non-uniformity and spatially normalized to the ICBM 152 Nonlinear Asymmetrical template version 2009c.^33^

Each run was preprocessed separately. Runs were co-registered to the T1-weighted image using one fMRI reference volume and slice-time corrected. Spatial smoothing was performed with an isotropic Gaussian kernel of 6 mm full width at half maximum. Motion artefacts were automatically and ‘non-aggressivley’^34^ removed using independent component analysis on the preprocessed images in Montreal Neurological Institute (MNI) space.^34^ Please see Benzait *et al*.^18^ for a more detailed description of the preprocessing pipeline of this dataset.

### First- and second-level general linear models

First- and second-level modelling of fMRI data was performed using SPM12 (www.fil.ion.ucl.ac.uk/spm). To remove slow signal drifts, a high-pass filter of 128 s was applied to each participant’s fMRI time series. To account for serial correlations resulting from unmodelled activity, an autoregressive model was applied. Non-steady-state outliers were excluded from the time series. Within-group second-level full factorial models were defined with two factors: regulation task (four levels: permit-negative, permit-neutral, downregulate-negative and upregulate-negative) and regulation phase (two levels: regulation and rest). For between-group comparisons, first-level contrasts of ‘upregulate-negative > permit-negative’, ‘downregulate-negative > permit-negative’ and ‘permit-negative > permit-neutral’ were used for the corresponding second-level tests. To account for demographical group differences, comparisons between HC and lTLE and rTLE patients were computed as ANCOVAs with age as a covariate of no interest. Comparisons between FLE and lTLE and rTLE patients were computed as ANCOVAs with age and age at epilepsy onset as covariates. *T*-tests were computed for comparisons between lTLE and rTLE patients and between HC and FLE patients.

Activation maps of within- and between-group comparisons were assessed for cluster-wise significance with an initial cluster-defining threshold of *P*_uncorr_ < 0.001 at the whole-brain level. The significance level for activation clusters was set to *P*_FWE_ < 0.05. To investigate the pattern of group differences, all groups were compared against each other, without correcting *P*-values for the number of between-group comparisons, as individual hypotheses were tested, i.e. each corresponding individual result has to be significant for each associated null hypothesis to be rejected.^35^ All peak activation coordinates are reported in MNI space, meaning sagittal slices with *x* > 0 are in the right hemisphere. Activation maps were visualized with MRIcroGL, version 1.2.20220720.^36^

### Time course analysis of regions of interest

#### Amygdala

Time courses were extracted from the left and right amygdala as defined by the Harvard-Oxford atlas using the nilearn module^37^ in Python.^38^ Detrended, temporally standardized and high-pass filtered trial time courses were averaged condition-wise across all four runs for each participant and baseline corrected using the first and last second. For within-group analyses, bootstrapped standard errors with 10 000 iterations and one-sample *t*-tests for differences between conditions were computed for each time point.

#### Clusters from voxel-based group comparisons

To further explore and validate results from voxel-based group comparisons, time courses were extracted from clusters in which groups differed and additional difference curves with bootstrapped 95% confidence intervals and two-sample *t*-tests, or ANCOVAs, were computed for each time point.

### Network-based whole-brain analysis

Task effects were profiled across seven resting-state networks^39^ as described in Caciagli *et al*.^19^ to investigate how the results from the voxel-based analysis align with functional brain organization. As brain hemispheres are differentially engaged during stimulus-based emotion processing^23^ and emotion regulation^17^ and the epileptogenic focus lateralization has been considered to affect such lateralized processing, hemispheres were analysed separately. The Schaefer parcellation scheme as implemented in nilearn^37^ was applied with a 200-parcel resolution.^40^ This parcellation allows the assignment of each parcel to one of seven resting-state networks. Individual beta weights for each parcel were extracted from contrasts that were significant in voxel-based between-group analyses. Networks that were engaged in any group were identified in an explorative analysis by first computing individual mean contrast weights per left and right network. Then, for each group, one-sample *t*-tests against zero were performed for each of the 14 networks with a significance level of *P*_uncorr_ < 0.05. Only for networks that showed a positive effect in any group, group differences were tested. *P*-values were false discovery rate (FDR) corrected for the number of networks submitted to between-group analyses. Additionally, *post hoc* analyses were conducted to establish whether networks submitted to between-group analyses were relevant for the respective task across groups with similar activation patterns. *P*-values were FDR corrected for the total number of networks (*n* = 14). Both FDR-corrected and uncorrected *P*-values are reported.

## Results

### Sample characteristics

Statistics for group demographics, epilepsy-related measures and self-reported depressiveness and state anxiety can be found in Table 1. HC and FLE patients were younger than lTLE patients [*F*(3,70) = 4.011, *P*_HC_ = 0.029, *P*_FLE_ = 0.053] and rTLE patients (*P*_HC_ = 0.006, *P*_FLE_ = 0.011). Age at epilepsy onset was lower in FLE than in rTLE patients [*F*(2,54) = 3.003, *P* = 0.019]. Both TLE groups did not differ regarding age (*P* = 0.464) or age at epilepsy onset (*P* = 0.345). Higher age and earlier epilepsy onset have been associated with reduced neural responses towards emotional stimuli, including in mesial temporal regions.^6,41^ Thus, both age and age at epilepsy onset were included as covariates in ANCOVAs comparing HC and TLE patients and FLE and TLE patients, respectively. Neither age nor age at epilepsy onset was correlated with state anxiety (*r*_age_ = −0.027, *P* = 0.841; *r*_epilepsy onset_ = −0.010, *P* = 0.943) or depression scores (*r*_age_ = −0.177, *P* = 0.205; *r*_epilepsy onset_ = 0.026, *P* = 0.855). Depression scores of lTLE patients were higher than those of controls and FLE patients [*F*(3,70) = 3.047, *P*_HC_ = 0.008, *P*_FLE_ = 0.022]. Also, state anxiety was higher in lTLE patients than in controls [*F*(3,70) = 2.221, *P*_lTLE_ = 0.020]. FLE and rTLE patients did not differ from controls regarding anxiety or depression (all *P* > 0.06). The number of antiseizure medications [*F*(2,54) = 1.737, all *P* > 0.08] and the number of patients treated with topiramate did not differ between groups [*P* = 0.372, Fisher’s exact test (FET)]. Antiseizure medication can have adverse cognitive effects,^42^ and topiramate may have particularly deteriorating effects.^43^ Education level was higher for controls than for FLE patients [*F*(3,68) = 2.449, *P* = 0.008]. There were no further group differences regarding education levels (all *P* > 0.09). Furthermore, there were no group differences regarding gender [*X*^2^ (3, *n* = 74) = 1.477, *P* = 0.688], handedness (*P* = 0.541, FET) or disease durations [*F*(2,54) = 0.227, all *P* > 0.52].

**Table 1:** State anxiety, Beck’s Depression Inventory scores, epilepsy-related characteristics and demographics for controls and patient groups

		HC		Left TLE		Right TLE		FLE	
		*M* (*SD*)	Min, max	*M* (*SD*)	Min, max	*M* (*SD*)	Min, max	*M* (*SD*)	Min, max
Age (y)		31.43 (9.09)	21.00, 52.00	39.41 (12.07)	20.04, 56.79	42.01 (12.41)	19.96, 62.32	32.48 (9.69)	18.81, 51.54
BDI		8.24 (5.62)	0.00, 23.00	17.10 (13.45)	0.00, 43.00	11.17 (8.86)	0.00, 34.00	9.67 (9.04)	0.00, 29.00
STAI-S		36.88 (7.53)	27.00, 55.00	45.71 (13.73)	25.00, 66.00	44.22 (10.71)	23.00, 60.00	40.58 (11.88)	22.00, 59.00
Age at epilepsy onset (y)				27.67 (15.32)	1.00, 54.00	32.28 (16.95)	9.00, 62.00	20.1 (12.5)	2.00, 45.00
Disease duration (y)				11.75 (15.67)	0.00, 53.79	9.73 (9.31)	0.32, 32.93	12.37 (10.39)	1.43, 37.54

				*Mdn* (*IQR*)	Min, Max	*Mdn* (*IQR*)	Min, Max	*Mdn* (*IQR*)	Min, Max
Number of ASMs				2.00 (1.00)	1.00, 3.00	2.00 (1.00)	1.00, 4.00	2.00 (1.00)	1.00, 4.00
		***N***		***n***		***n***		***n***	
Sex	Male	11		15		10		13	
	Female	6		6		8		5	
Education	No degree	/		1		/		/	
	Basic secondary schooling (9 y)	1		3		3		6	
	Secondary education (10 y)	7		10		10		9	
	University entrance diploma (12 or 13 y)	9		6		5		3	
	Unknown	/		1		/		/	
Handedness	Left	2		5		2		1	
	Right	14		16		16		15	
	Ambidexter	1		/		/		1	
	Unknown	/		/		/		1	
Neural antibodies	GAD	/		1		2		/	
	LGI1	/		/		2		/	

HC, healthy controls; TLE, temporal lobe epilepsy; FLE, frontal lobe epilepsy; *M*, mean; *SD*, standard deviation of the mean; *Mdn*, median; *IQR*, interquartile range; y, years; Min, minimal value; Max, maximal value; BDI, Beck’s Depression Inventory; STAI-S, state subscale from the state-trait anxiety inventory; ASMs, antiseizure medication; GAD, glutamic acid decarboxylase; LGI1, leucine-rich glioma inactivated 1.

Two lTLE patients had a short focal seizure some minutes before their fMRI scanning session. Afterwards, both felt ready for the fMRI task and did not present any signs of postictal impairments. While postictal cortical blood flow alterations may affect fMRI activations, comparisons between within-group results excluding or including their data did not suggest marked differences regarding activation patterns (see Supplementary Figs. 1 and 2). Thus, their data were included for the following analyses. In the rTLE group, one patient was blind in the left eye. Again, group results were not substantially altered by including or excluding this patient (see Supplementary Fig. 3).

### Behavioural results

Participants were instructed to up- or downregulate their emotions towards negative pictures or to permit them towards negative or neutral pictures. After each trial, participants were asked to rate their negative affect intensity on a 7-point Likert scale, ranging from 1 = ‘very weak’ to 7 = ‘very strong’. Across groups, self-reported negative affect differed as expected by task [*F*(3,219) = 189.148, all *P*_Bonferroni_ < 0.001]. It was lowest for neutral images (*M* = 2.21, *SD* = 0.89). Downregulating emotions resulted in lower negative affect (*M* = 3.16, *SD* = 1.15) than permitting emotions (*M* = 4.47, *SD* = 1.03), while upregulating emotions resulted in the most negative affect (*M* = 5.42, *SD* = 1.02). Groups did not differ overall in their ratings, nor was there an interaction between group and task condition (*P*_uncorr_ > 0.10 for all main effects of group and all interaction effects of group × condition; see Supplementary Fig. 4).

### Functional MRI results

#### Stimulus-based processing of negative versus neutral pictures

##### Voxel-based analyses—within groups

**Figure 2:**
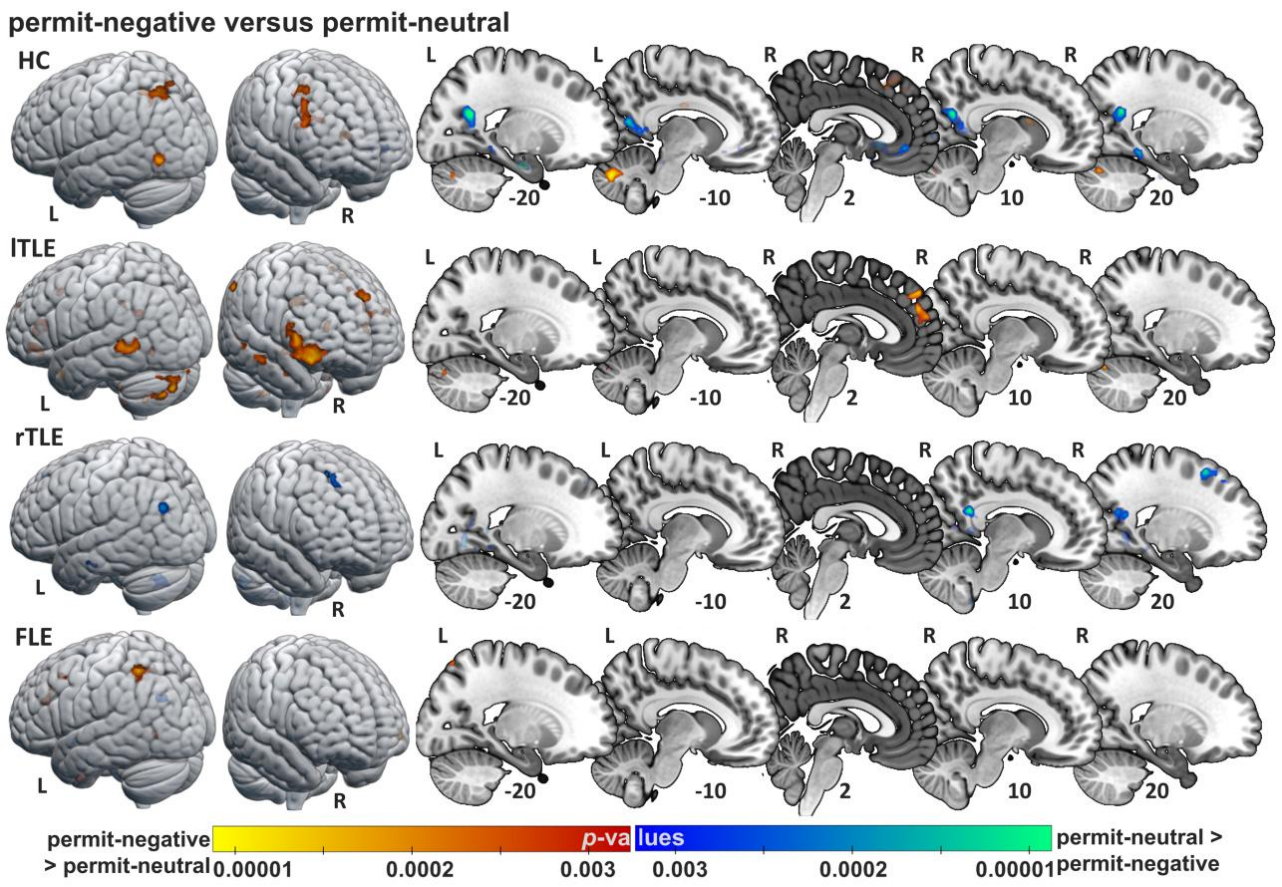
**Whole-brain activations for permitting emotions towards negative versus neutral images.** To illustrate larger patterns, activation maps are shown at *P*_uncorr_ < 0.001 and *P*_uncorr_ < 0.005 (more transparent) with an extent threshold of 50 voxels for display purposes. Activation maps show within-group *t*-contrasts. Random field theory-based corrections, as implemented in SPM12 (www.fil.ion.ucl.ac.uk/spm), were applied on the cluster level to control the FWE rate at *P*_FWE_ < 0.05. L = left, R = right, HC = healthy controls, lTLE = left temporal lobe epilepsy, rTLE = right temporal lobe epilepsy, FLE = frontal lobe epilepsy. For permit-negative > permit-neutral, controls (*n* = 17) displayed increased right frontal and left cerebellar activity. For permit-neutral > permit-negative, they showed activity in the bilateral posterior medial temporal regions and in the precuneus. lTLE patients (*n* = 21) displayed distributed mostly right-sided inferior and dorsomedial prefrontal, bilateral temporo-occipital and cerebellar activity for permit-negative > permit-neutral. They did not show any activations in the opposite contrast. On the contrary, rTLE patients (*n* = 18) displayed increased activity only for permit-neutral > permit-negative, which included the right superior frontal gyrus and right precuneus and left posterior medial temporal regions. FLE patients (*n* = 18) displayed activity in the left superior parietal lobe for permit-negative > permit-neutral but no enhanced activations in the opposite contrast.

Except for rTLE patients, all groups displayed significant clusters for permitting emotions towards negative compared to neutral images (see Fig. 2 and Supplementary Table 2). In controls, activity was enhanced in the right middle and inferior frontal gyrus (*k* = 150, *P* = 0.010) and in the left cerebellum (*k* = 129, *P* = 0.021). In lTLE patients, activations in this contrast were descriptively more distributed than in controls and were mostly right sided, including the inferior frontal gyrus, orbitofrontal cortex and frontal pole (*k* = 777, *P* < 0.001), dorsomedial prefrontal cortex (*k* = 447, *P* < 0.001), bilateral temporo-occipital regions (*k*_Left_ = 206, *P*_Left_ = 0.002; *k*_Right_ = 131, *P*_Right_ = 0.023) and cerebellum (*k*_Left_ = 389, *P*_Left_ < 0.001; *k*_Right1_ = 156, *P*_Right1_ = 0.013; *k*_Right2_ = 123, *P*_Right2_ = 0.030). In FLE patients, the left superior parietal lobe was activated (*k* = 147, *P* = 0.012). For the contrast permit-neutral > permit-negative, controls displayed bilateral activations including the precuneus (*k_Left_* = 367, *P*_Left_ < 0.001; *k*_Right_ = 239, *P*_Right_ = 0.001) and posterior medial temporal regions encompassing the left fusiform cortex, bilateral lingual and parahippocampal gyrus (*k*_Left_ = 204, *P*_Left_ = 0.002; *k*_Right_ = 148, *P*_Right_ = 0.010). rTLE patients displayed activations in the right superior frontal gyrus (*k* = 107, *P* = 0.050), right precuneus (*k* = 157, *P* = 0.008) and left lingual and parahippocampal gyrus (*k* = 235, *P* = 0.001), whereas lTLE and FLE patients showed no significant activations in this contrast.

##### Voxel-based analyses—between groups

**Figure 3:**
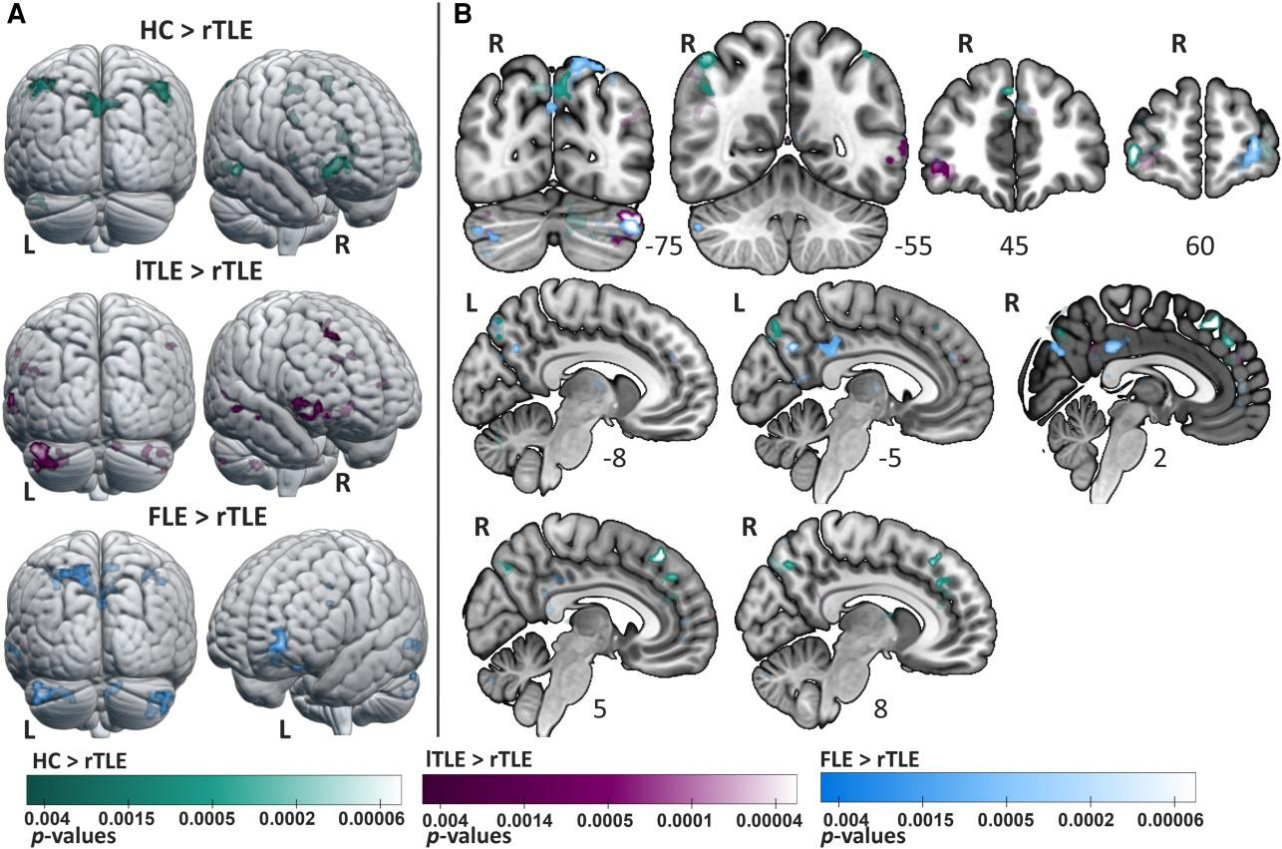
**Group comparisons for permitting emotions towards negative versus neutral images.** Activation maps show between-group *t*-contrasts of controls (green), lTLE (magenta) and FLE patients (blue) > rTLE patients projected on individual 3D brain surfaces (**A**) and on the same MNI template (**B**). To illustrate larger patterns, activation maps are shown at *P*_uncorr_ < 0.001 and *P*_uncorr_ < 0.005 with an extent threshold of 50 voxels for display purposes. More transparent maps show *P*_uncorr_ < 0.005 activations in both panels. Colour bars show *P*-values for the different group comparisons. L = left, R = right, HC = healthy controls, lTLE = left temporal lobe epilepsy, rTLE = right temporal lobe epilepsy, FLE = frontal lobe epilepsy. HC (*n* = 17), lTLE (*n* = 21) and FLE patients (*n* = 18) displayed higher activity than rTLE patients (*n* = 18) for permitting emotions towards negative compared to neutral scenes in distributed regions, including the prefrontal and parietal cortices. Random field theory-based corrections, as implemented in SPM12 (www.fil.ion.ucl.ac.uk/spm), were applied on the cluster level to control the FWE rate at *P*_FWE_ < 0.05.

Contrasting the conditions permit-negative > permit-neutral between groups, controls displayed higher activity than rTLE patients in distributed, mostly right-sided regions, encompassing the right superior frontal gyrus and frontal pole, bilateral angular gyrus and precuneus (see Fig. 3 and Table 2 for all group comparisons; for time courses, see also Supplementary Figs. 5–7). For lTLE > rTLE, a partly similar pattern emerged, including the right superior frontal gyrus and frontal pole and the left cerebellum. Note that in HC, the right angular gyrus and right precuneus and, in lTLE patients, the right superior frontal gyrus showed stronger deactivation in response to neutral compared to negative pictures. FLE patients displayed higher activations for negative compared to neutral pictures than rTLE patients in the left frontal pole, bilateral cerebellum and posterior cingulate cortex, which was slightly more deactivated for neutral pictures in FLE and for negative pictures in rTLE patients. However, reviewing posterior cingulate cortex time courses did not reveal group differences between rTLE and FLE patients, which might be related to averaging over the whole cluster. In summary, rTLE patients displayed a pattern of diminished prefrontal and posterior activation compared to the other three groups during stimulus-based processing of negative compared to neutral images. Moreover, HC displayed less deactivation for neutral than for negative pictures in the left ventral posterior precuneus compared to FLE patients.

**Table 2:** Between-group comparisons for contrasting permit-negative > permit-neutral

Side	Activation peak	X	Y	Z	*z*-score	Volume (voxels)	*P*_FWE_ (cluster)
HC > rTLE							
R	Superior frontal gyrus	6	36	52	4.46	110	0.011
R	"	10	30	46	4.07		
R	Frontal pole/orbitofrontal cortex	40	60	−2	4.33	119	0.007
R	"	28	60	0	3.47		
R	"	32	64	−6	3.23		
R	Angular gyrus	44	−56	56	3.97	178	0.001
R	"	46	−58	44	3.91		
R	"	44	−52	36	3.26		
R	Precuneus	10	−76	46	3.79	156	0.001
R	"	10	−66	46	3.68		
L	"	−6	−76	52	3.68		
L	Angular gyrus	−38	−64	60	4.99	95	0.023
L	Supramarginal gyrus	−44	−52	60	3.62		
L	Angular gyrus	−48	−62	52	3.27		
lTLE > rTLE							
R	Superior frontal gyrus	24	30	48	4.15	93	0.034
R	"	20	24	52	3.92		
R	Frontal pole/orbitofrontal cortex	44	44	−4	3.75	101	0.023
R	"	54	40	−12	3.58		
R	"	30	38	−10	3.58		
L	Cerebellum	−48	−72	−28	4.99	292	<0.001
L	"	−40	−74	−26	4.31		
L	"	−48	−68	−40	4.31		
FLE > rTLE							
L	Frontal pole/orbitofrontal cortex	−32	64	−4	4.08	119	0.008
L	"	−28	60	2	3.90		
L	"	−28	56	−8	3.50		
L	Posterior cingulate cortex	−2	−34	38	3.76	123	0.006
L	"	−2	−46	36	3.57		
R	Cerebellum	36	−70	−36	4.25	226	<0.001
R	"	46	−68	−36	4.19		
R	"	52	−56	−38	3.90		
L	Cerebellum	−44	−76	−32	4.62	117	0.008
L	"	−48	−70	−28	4.15		
L	"	−48	−70	−40	3.96		
FLE > HC							
L	Precuneus	−16	−58	22	4.51	186	0.001
L	"	−8	−58	12	4.27		
L	"	−16	−62	30	3.22		

L, left; R, right; HC, healthy controls; lTLE, left temporal lobe epilepsy; rTLE, right temporal lobe epilepsy; FLE, frontal lobe epilepsy.

##### Network-based analyses

**Figure 4:**
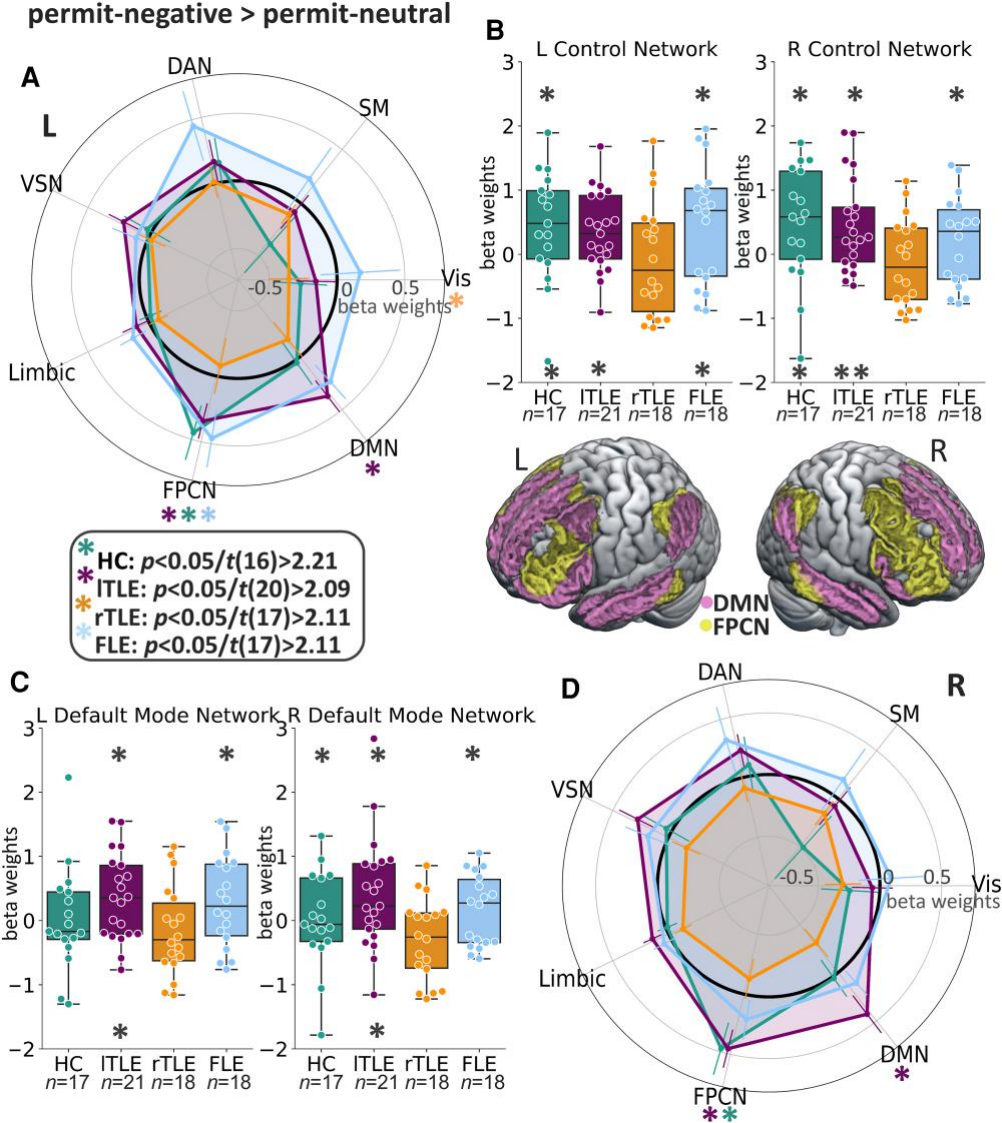
**Network-based analyses for stimulus-based processing of negative versus neutral pictures.** (**A** and **D**) Spider charts show mean beta weights of controls (green), FLE (blue), lTLE (magenta) and rTLE (orange) patients in seven left (**A**) and right (**D**) resting-state networks. Error bars represent standard errors. The bold black circle marks the zero line. Asterisks denote significant (*P*_uncorr_ < 0.05) group mean deviations from zero as revealed by one-sample *t*-tests. Corresponding test statistics are detailed in the main text. (**B** and **C**) Boxplots display group activations in the left and right frontoparietal control network (FPCN; **B**) and default mode network (DMN; **C**). In **B** and **C**, dots represent individual participants. The *y*-axes show beta weights. Asterisks below boxplots denote significant (*P*_uncorr_ < 0.05) group mean deviations from zero as revealed by one-sample *t*-tests. Asterisks above boxplots denote higher (*P*_FDR_ < 0.05) group activations compared to rTLE patients. To account for demographical group differences, comparisons between HC and rTLE patients were computed as ANCOVAs with age as a covariate of no interest. Comparisons between FLE and rTLE patients were computed as ANCOVAs with age and age at epilepsy onset as covariates. *T*-tests were computed for comparisons between left and right TLE patients. Corresponding test statistics are detailed in the main text. **P < 0.01, *P < 0.05; DAN = dorsal attention network, SM = somatomotor network, Vis = visual network, VSN = ventral salience network; L = left, R = right; HC = healthy controls, lTLE = left temporal lobe epilepsy, rTLE = right temporal lobe epilepsy, FLE = frontal lobe epilepsy. All groups, except rTLE patients (*n* = 18), displayed enhanced activity for permitting emotions towards negative compared to neutral pictures in the FPCN. HC (*n* = 17), lTLE (*n* = 21) and FLE patients (*n* = 18) showed higher activity in this contrast than rTLE patients in the FPCN and in the DMN.

Testing averaged network activation against zero in each group revealed that HC, FLE and lTLE patients displayed frontoparietal control network (FPCN) activation during stimulus-based processing of emotions towards aversive versus neutral scenes (see Fig. 4). While FPCN activation was bilateral for HC [*t*_left_(16) = 2.15, *P*_uncorr_ = 0.047; *t*_right_(16) = 2.16, *P*_uncorr_ = 0.046] and lTLE patients [*t*_left_(20) = 2.58, *P*_uncorr_ = 0.018; *t*_right_(20) = 2.89, *P*_uncorr_ = 0.009], for FLE patients, it was left sided [*t*(17) = 2.11, *P*_uncorr_ = 0.050). Additionally, lTLE patients displayed bilateral default mode network (DMN) engagement [*t*_left_(20) = 2.42, *P*_uncorr_ = 0.025; *t*_right_(20) = 2.15, *P*_uncorr_ = 0.044]. Conversely, rTLE patients did not display enhanced activity in any network for permit-negative > permit-neutral. Instead, they showed enhanced left visual network activation in the opposite contrast [*t*(17) = 2.37, *P*_uncorr_ = 0.030].

Since bilateral FPCN and DMN were the only networks more engaged during negative stimulus-based emotion processing than during the neutral condition, group comparisons for the contrast permit-negative > permit-neutral were computed for these networks. All groups were compared against each other. Compared to rTLE patients, FLE patients and HC displayed higher bilateral FPCN activity [*F*_FLE,left_(1,32) = 5.92, *P*_uncorr/FDR_ = 0.021/0.028; *F*_FLEright_(1,32) = 4.46, *P*_uncorr/FDR_ = 0.043/0.043; *F*_HC,left_(1,32) = 7.71, *P*_uncorr/FDR_ = 0.009/0.018; *F*_HCright_(1,32) = 10.71, *P*_uncorr/FDR_ = 0.003/0.012]. lTLE patients displayed higher activity than rTLE patients only in the right FPCN [*t*(37) = 2.60, *P*_uncorr/FDR_ = 0.013/0.026]. Moreover, HC, lTLE and FLE patients displayed higher right DMN activation than rTLE patients [*F*_HC_(1,32) = 5.45, *P*_uncorr/FDR_ = 0.026/0.035; *t*_lTLE_(37) = 2.92, *P*_uncorr/FDR_ = 0.006/0.024; *F*_FLE_(1,32) = 8.17, *P*_uncorr/FDR_ =0.007/0.028]. lTLE and FLE patients also displayed higher left DMN activation than rTLE patients [*t*_lTLE_(37) = 2.31, *P*_uncorr/FDR_ = 0.027/0.036; *F*_FLE_(1,32) = 6.82, *P*_uncorr/FDR_ = 0.014/0.028]. No other group comparisons were significant.

#### Regulatory versus stimulus-based processing of negative pictures

##### Voxel-based analyses—within groups

**Figure 5:**
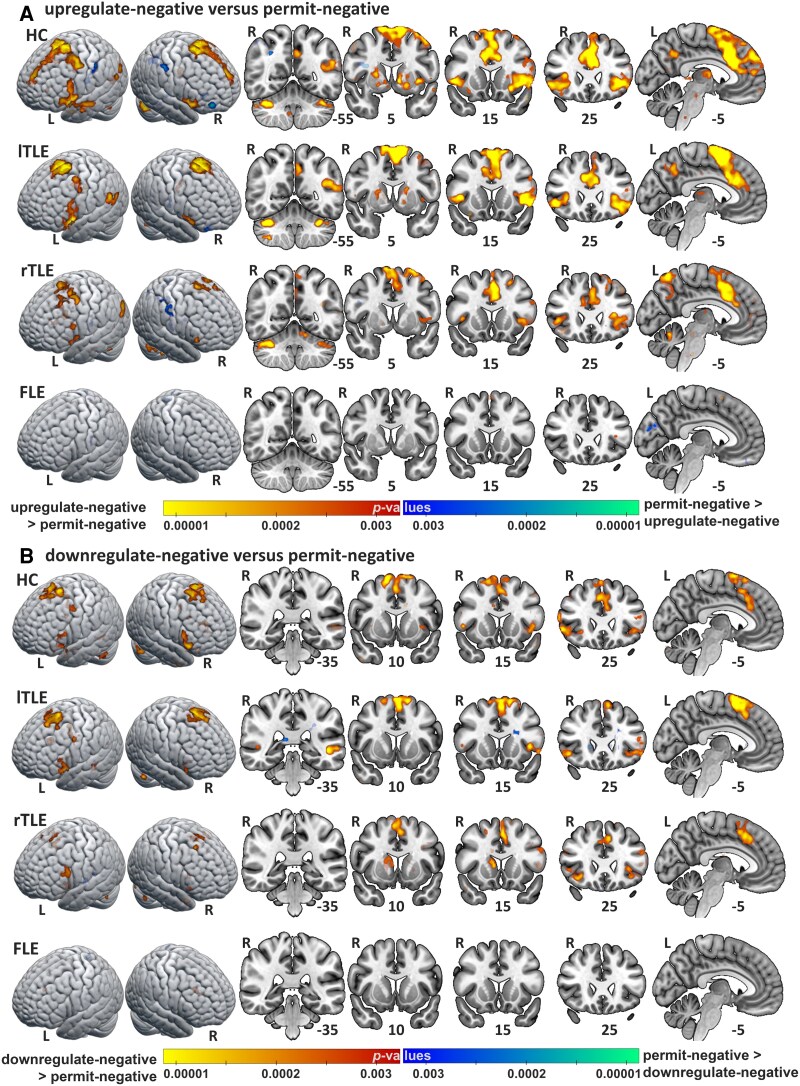
**Upregulate-negative > permit-negative (A) and downregulate-negative > permit-negative (B) activation maps in left (L) and right (R) temporal lobe epilepsy (TLE) patients.** To illustrate larger patterns, activation maps are shown at *P*_uncorr_ < 0.001 and *P*_uncorr_ < 0.005 (more transparent) with an extent threshold of 50 voxels for display purposes. Activation maps show within-group *t*-contrasts. L = left, R = right, HC = healthy controls. Controls (*n* = 17) and left (*n* = 21) and right TLE patients (*n* = 18) displayed highly similar activation maps during upregulation and downregulating emotions compared to permitting emotions towards negative pictures. Frontal lobe epilepsy (FLE) patients (*n* = 18) did not show significant activity differences in these contrasts. Random field theory-based corrections, as implemented in SPM12 (www.fil.ion.ucl.ac.uk/spm), were applied on the cluster level to control the FWE rate at *P*_FWE_ < 0.05.

Whole-brain activations for upregulate-negative > permit-negative and downregulate-negative > permit-negative are shown in Fig. 5 and listed in Supplementary Table 2. Contrasting regulating to permitting emotions towards negative pictures revealed activity in the dorsomedial and ventrolateral prefrontal cortices, posterior brain regions like the left lateral temporal lobe, left angular gyrus and precuneus and also subcortical striatal regions and the cerebellum. Reappraisal-related activations in HC, rTLE and lTLE patients showed substantial overlap and replicated extensive former fMRI research on emotion regulation through reappraisal in healthy participants.^5,17^ FLE patients did not display any reappraisal-related activity differences, even at a low cluster-forming threshold (for *P*_uncorr_ < 0.01, all *P*_FWE_ > 0.05; see Benzait *et al*.^18^).

##### Voxel-based analyses—between groups

**Figure 6:**
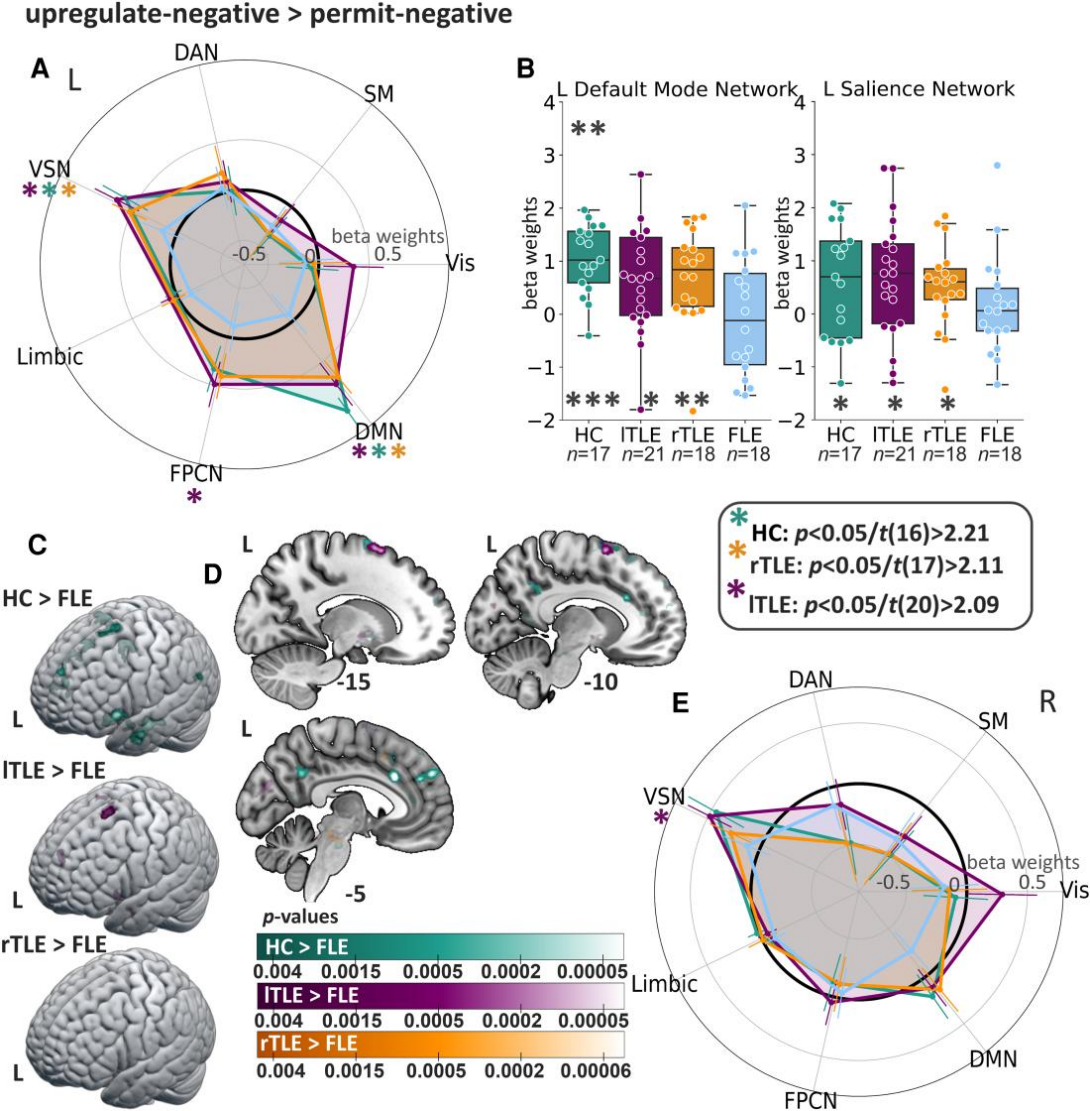
**Network- and voxel-based analyses for upregulate-negative versus permit-negative.** (**A** and **E**) Spider charts show mean beta weights of controls (green), FLE (blue), lTLE (magenta) and rTLE (orange) patients in seven left (**A**) and right (**E**) resting-state networks. Error bars represent standard errors. The bold black circle marks the zero line. Asterisks denote significant (*P*_uncorr_ < 0.05) group mean deviations from zero as revealed by one-sample *t*-tests. Corresponding test statistics are detailed in the main text. (**B**) Boxplots display group activations in the left default mode network (DMN) and ventral salience network (VSN). Dots represent individual participants. The *y*-axes show beta weights. Asterisks below boxplots denote significant (*P*_uncorr_ < 0.05) group mean deviations from zero as revealed by one-sample *t*-tests. Asterisks above boxplots denote higher (*P*_FDR_ < 0.05) group activations compared to FLE patients. To account for demographical group differences, comparisons between FLE and left and right TLE patients were computed as ANCOVAs with age and age at epilepsy onset as covariates. *T*-tests were computed for comparisons between HC and FLE patients. Corresponding test statistics are detailed in the main text. Except for FLE patients (*n* = 18), all groups displayed enhanced activity for upregulating compared to permitting negative emotions in the left DMN and VSN. HC (*n* = 17) and lTLE patients (*n* = 21) showed higher activity in this contrast than FLE patients (*n* = 18) in parts of the left DMN. (**C** and **D**) Activation maps show between-group *t*-contrasts, as implemented in SPM12 (www.fil.ion.ucl.ac.uk/spm), of controls (green), lTLE (magenta) and rTLE patients (orange) > FLE patients projected on individual 3D brain surfaces (**C**) and on the same MNI template (**D**). To illustrate larger patterns, activation maps are shown at *P*_uncorr_ < 0.001 and *P*_uncorr_ < 0.005 with an extent threshold of 50 voxels for display purposes. More transparent maps show *P*_uncorr_ < 0.005 activations. Colour bars show *P*-values for the different group comparisons. SPM12’s random field theory-based corrections were applied on the cluster level to control the FWE rate at *P*_FWE_ < 0.05. Note that no significant group differences were found for rTLE > FLE patients. ***P < 0.001, **P < 0.01, *P < 0.05; DAN = dorsal attention network, SM = somatomotor network, Vis = visual network, FPCN = frontoparietal control network; L = left, R = right; HC = healthy controls, lTLE = left temporal lobe epilepsy, rTLE = right temporal lobe epilepsy, FLE = frontal lobe epilepsy.

Left and right TLE patients did not differ from HC or from each other during up- or downregulating emotions. As previously reported and shown in Fig. 6, HC displayed higher activity during upregulating compared to permitting negative emotions than FLE patients in distributed left-sided regions, including the inferior and superior frontal gyrus, anterior cingulate cortex, angular gyrus and middle temporal gyrus.^18^ lTLE patients showed higher upregulation-related activity than FLE patients in the left superior frontal gyrus (see Fig. 6; peak = −12, 12, 64; *z*_peak_ = 3.93, *k* = 99, *P*_cluster(FWE)_ = 0.047). rTLE patients did not differ significantly from FLE patients. There were no group differences for contrasting downregulate-negative > permit-negative.

##### Network-based analyses

Contrasting upregulating with permitting negative emotions, HC, rTLE and lTLE patients displayed left DMN [*t*_HC_(16) = 6.52, *P*_uncorr_ < 0.001; *t*_rTLE_(17) = 3.40, *P*_uncorr_ = 0.003; *t*_lTLE_(20) = 2.84, *P*_uncorr_ = 0.010] and ventral salience network (VSN) activation [*t*_HC_(16) = 2.41, *P*_uncorr_ = 0.029; *t*_rTLE_(17) = 2.75, *P*_uncorr_ = 0.014; *t*_lTLE_(20) = 2.78, *P*_uncorr_ = 0.012; see Fig. 6]. lTLE patients additionally displayed left FPCN [*t*(20) = 2.10, *P*_uncorr_ = 0.048] and right VSN activity [*t*(20) = 2.42, *P*_uncorr_ = 0.025], whereas FLE patients did not show enhanced activity in any network. Group comparisons were computed for the left DMN and FPCN and bilateral VSN. HC and lTLE patients displayed higher left DMN activity than FLE patients for upregulating compared to permitting emotions, which, for lTLE patients, did not survive FDR correction [*t*_HC_(33) = 3.62, *P*_uncorr/FDR_ < 0.001/0.004; *F*_lTLE_(1,35) = 4.58, *P*_uncorr/FDR_ = 0.039/0.107]. There were no other significant group differences.

Taken together, regulation-related activation patterns of TLE patients were highly similar to those of HC in the voxel-based as well as in the network-based analyses. Accordingly, there were no significant differences between TLE patients and HC. By contrast, FLE patients did not present regulation-related activity and displayed significantly decreased activations compared to lTLE patients and HC for upregulating compared to permitting emotions.

#### *Post hoc* network analyses across groups

To establish whether the networks submitted to between-group analyses were relevant for the respective task across groups, *post hoc* network analyses were conducted across groups. For permit-negative > permit-neutral, *t*-tests were computed across HC, lTLE and FLE patients (see the Materials and methods, Network-based whole-brain analysis section). These analyses revealed bilateral FPCN activations [*t*_left_(55) = 3.94, *P*_uncorr/FDR_ < 0.001/0.003; *t*_right_(55) = 3.68, *P*_uncorr/FDR_ < 0.001/0.004], while DMN activation did not survive FDR correction [*t*_left_(55) = 2.37, *P*_uncorr/FDR_ = 0.021/0.098; *t*_right_(55) = 2.09, *P*_uncorr/FDR_ = 0.041/0.115]. For upregulate-negative > permit-negative, t-tests were computed across HC, lTLE and rTLE patients. These analyses revealed activity in the left FPCN [*t*(55) = 3.12, *P*_uncorr/FDR_ = 0.003/0.011], left DMN [*t*(55) = 6.42, *P*_uncorr/FDR_ < 0.001/ < 0.001] and bilateral VSN [*t*_left_(55) = 4.59, *P*_uncorr/FDR_ < 0.001/ < 0.001; *t*_right_(55) = 3.32, *P*_uncorr/FDR_ = 0.002/0.009]. Thus, except for DMN engagement during stimulus-based processing of negative versus neutral pictures, which did not survive FDR correction, the relevance of all networks submitted to between-group analyses was confirmed in *post hoc* analyses across groups.

#### Amygdala time course analysis

The amygdala plays a well-established role in emotion processing and is a major target of emotion regulation efforts.^4,5^ Given numerous findings of diminished ipsilesional amygdala responses in TLE patients,^6-9^ amygdala time courses were explored in the four experimental conditions. During regulation, we found bilateral task-related amygdala modulations in controls and TLE patients (see Supplementary Fig. 8). As reported previously,^18^ FLE patients displayed such modulations only in the right amygdala. No group showed a significant difference for permit-negative versus permit-neutral. Descriptively, time course heights appeared to be reduced in the left amygdala for lTLE patients and bilaterally for rTLE patients compared to HC.

## Discussion

Emotion regulation, an issue of high clinical importance,^1^ has barely been investigated in epilepsy. TLE patients have previously reported more emotion regulation difficulties than HC^16^ and have displayed functional alterations in brain regions central to emotion processing and regulation. Investigating neural substrates of reappraisal in TLE could inform psychological treatment approaches, as reappraisal is key to cognitive behavioural therapy and is associated with psychological well-being and personal resilience.^44^ Additionally, altered stimulus-based emotion processing may impair adaptive behaviour in emotional situations and may interact with emotion regulation. Therefore, we investigated emotion regulation and processing in left and right TLE patients. Moreover, they were compared to FLE patients to further our understanding of differences and commonalities between different types of focal epilepsies.

On the behavioural level, all groups reported increased negative affect in response to negative compared to neutral pictures and successful up- and downregulation of their negative affect, indicating adequate task performance. Affect ratings did not differ between groups. However, such results may be confounded by potential group-specific differences in individual reference systems for emotional experiences.^45^ Moreover, self-reports may also reflect social desirability, leaving them difficult to interpret. Due to such confounding factors, neural activation might be a valuable complementary and more specific measure to study the impact of focal epilepsy on emotion regulation. Indeed, the present fMRI data revealed considerable task-dependent differences between groups.

On the neural level, within-group analyses revealed mostly frontal and/or parietal activations during stimulus-based processing of negative compared to neutral scenes in all groups but rTLE patients. In corresponding network analyses, HC, lTLE and FLE patients showed increased FPCN activity. In contrast to previous studies^46,47^ that compared processing of negative to neutral pictures, our analyses revealed few emotion effects in visual cortices. For controls and for rTLE patients in particular, visual regions showed an inverse emotion effect. This could be due to the three-to-one ratio of negative to neutral pictures, as visual regions have been shown to display decreased activity with repeated stimulus presentation.^48,49^ Furthermore, stronger repetition suppression has been found for negative than for neutral stimuli.^50^ While, in our study, there was no exact stimulus repetition, negative images often featured similar content (e.g. car accidents and guns). Moreover, in our study, the analysed trial phase followed a short affect induction phase where the stimulus was presented without instructions. This approach ensured that participants were familiar with the images before they were asked to regulate or permit their feelings, allowing the analyses to focus on brain regions involved in sustained emotion processing, rather than initial visual or emotional responses.

Group comparisons for stimulus-based emotion processing revealed enhanced neural responses for controls, lTLE and FLE patients compared to rTLE patients, both in the voxel-based and in the subsequent network-based analyses. We found overall reduced DMN and FPCN activity for rTLE patients compared to all other groups, particularly in prefrontal and parietal regions. No differences were found between lTLE and FLE patients or HC. Of note, only lTLE patients showed DMN engagement during stimulus-based emotion processing. FPCN and DMN have been shown to be positively coupled during various tasks and are both associated with mentalizing,^51-53^ emotion processing,^46,52,53^ empathy^54^ and emotional awareness.^51,55^

Frontal and parietal control regions have been repeatedly associated with emotion processing.^46,47,52,55-58^ In the prefrontal cortex, the same regions may be implicated in emotion generation and regulation.^55,59^ The superior frontal gyrus (HC and lTLE > rTLE) has been shown to be involved in emotion regulation^55^ and, along with parietal midline regions, self-referential emotion processing.^60^ The FPCN, particularly its lateral frontopolar part (all groups > rTLE), has been associated with meta-cognitive awareness of thoughts and emotions,^55,61,62^ working in concert with the DMN.^52,61^ Consistent with a role of the FPCN in emotional awareness, FPCN and DMN regions have been associated with self-reports of perceived polarity and intensity of emotional experiences during watching movie scenes.^8,57^ As outlined above, emotion effects presented here represent sustained emotion processing, which presumably involves self-awareness of feelings, especially in the context of an emotion regulation task. Interestingly, in the context of memory performance, rTLE patients have been shown to display reduced self-awareness compared to controls and lTLE patients,^63^ which aligns with a prioritized role of the right hemisphere in self-awareness.^64^ While the present results may thus reflect altered emotional self-awareness in rTLE, a further interpretation might be that, in line with a presumed instrumental role of the right amygdala in emotion processing,^12,15^ emotional responsiveness is reduced in rTLE. Reduced fear intensity ratings have previously been found for rTLE but not for lTLE patients compared to controls.^8^ In line with this, it has been reported that rTLE patients suffer less frequently from psychopathologies like depression, anxiety and psychosis than lTLE patients.^65^ Moreover, fear intensity ratings have been found to be positively correlated with activity in FPCN and DMN regions.^8^ The present results indicate that in rTLE patients, neuronal responsiveness towards negative emotional cues is reduced in these networks. If indicative of reduced emotional responsiveness, this might be protective against certain psychopathologies in rTLE. Given that at present we did not observe behavioural differences, this conclusion is speculative. However, our findings highlight the importance of further research in this field to get a sound picture of how different epilepsy syndromes impact functional activations during different tasks and how this relates to behavioural and psychopathological measures.

FLE patients displayed less deactivation than HC in response to negative pictures in the left precuneus. Notably, this DMN region was deactivated during negative and neutral permit trials in all groups except FLE. For HC and rTLE patients, this deactivation was stronger for negative than for neutral pictures. This pattern is in line with the notion that medial parietal deactivation is particularly sensitive to externally cued cognitive processes,^66-68^ which should be more pronounced during negative scene perception as negative stimuli capture attention more than neutral ones^69^ and may prompt spontaneous implicit emotion regulation. Consistent with the reduced precuneus deactivation in FLE patients reported here, disrupted DMN deactivation has previously been observed for FLE patients compared to controls^70^ and TLE patients^19^ in cognitive tasks. The present results suggest that DMN deactivation in FLE is, at least in part, impaired in a variety of tasks.

Contrary to the present results of altered stimulus-based emotion processing in rTLE, a recent fMRI study^71^ reported largely preserved activations for negative compared with neutral pictures in post-resectional rTLE patients. EEG data from the same sample, however, showed diminished right-sided emotional modulation for rTLE patients, particularly during early processing.^14^ These group differences were reduced in the later processing stages, possibly reflecting compensational influences.^14^ Such potential compensation might be impeded by ongoing epileptic activity,^27^ which may explain why rTLE patients in our study displayed disrupted stimulus-based emotion processing.

This study, for the first time, directly compared TLE to FLE patients during stimulus-based emotion processing and emotion regulation and linked group differences to large-scale network disruptions, enabling us to establish differences and commonalities between those focal epilepsy syndromes on the neural level, advancing clinical knowledge about the specific impacts of epilepsy focus localization.^19,28,72^ This is particularly relevant as behavioural measures may be insufficient to capture existing impairments in epilepsy patients.^45,73^ So far, they inconsistently distinguish focal epilepsy syndromes.^28^ Moreover, our study was the first to investigate the neural substrates of emotion regulation in TLE. In contrast to findings of distributed alterations in rTLE patients during stimulus-based processing, overall activation patterns during both up- and downregulation of emotions were highly similar between HC, rTLE and lTLE patients, suggesting largely intact neural emotion regulation circuits in TLE. Also, lTLE patients exhibited higher activations than FLE patients during upregulating emotions, albeit to a lesser extent. For HC, lTLE and rTLE patients, the left DMN and VSN displayed higher activations during upregulating than permitting emotions. Both networks are, along with the FPCN, commonly engaged during emotion regulation through reappraisal.^3,74^ DMN activity may reflect increased internally directed cognitive efforts, like mentalizing or imagery.^51^ Furthermore, the left DMN is involved in semantic processing and semantic control.^68^ Thus, our results suggest that a frontal epilepsy focus seems to impede the recruitment of prefrontal and posterior regions during emotion regulation, whereas a temporal epilepsy focus has less severe consequences for neural circuits underlying emotion regulation, including temporal semantic processing regions. Furthermore, our results indicate that previous findings of impaired neural responses in TLE patients during semantic language tasks^19,22^ may not be generalizable to reappraisal, despite its semantic task demands. However, although the present samples are similar to or larger than in most previous studies,^6,8,13,29,75,76^ they allow us to detect only relatively large effects at an adequate power level. Therefore, more subtle regulation-related changes may still exist.

Consistent with preserved activations during emotion regulation, like HC, TLE patients displayed bilateral regulation-related amygdala modulations, indicating that regulation efforts successfully target the ipsilesional amygdala. While the present study showed that regulation-related modulations in the left amygdala were absent in FLE patients as a whole, future studies should clarify the role of focus lateralization in this group. Overall, our results underline that although epilepsy is a network disorder with widespread functional disruptions,^25-27^ the specific site of the epileptic focus has a significant impact on cognitive and emotional processes.

Results of altered stimulus-based emotion processing in rTLE but well-preserved activation patterns during emotion regulation suggest that rTLE patients—although impaired in their spontaneous reactions to at least negative emotional stimuli—are able to intentionally generate adequate emotional responses, even inducing modulations of amygdala activity. These new insights underline the potential of cognitive behavioural therapy for treating psychological disorders in TLE patients, as this approach uses reappraisal as a core coping strategy.^2^

Moreover, the present results show that in the same group, a network that is disrupted in one task may be adequately activated in another task. Thus, our data clearly indicate that disrupted network recruitment is not simply a function of epileptic focus localization. Instead, it dynamically changes with task demands as well.

In sum, emotion regulation was found to be impaired in FLE but not in lTLE or rTLE. Conversely, stimulus-based emotion processing was found to be impaired in rTLE but largely intact in FLE. These findings point to a double dissociation between rTLE and FLE, shedding new light on the issue of lateralized emotion processing and how it interacts with epileptic focus localization. Moreover, while standard neuropsychological measures may have limited utility in distinguishing focal epilepsy syndromes,^19^ the present research highlights the potential of exploring neural activation patterns during cognitive and emotional processes for diagnostic purposes. This can inform more tailored treatment strategies and, ultimately, improve the quality of life for epilepsy patients.

## Supplementary Material

fcag270_Supplementary_Data

## Data Availability

The data that support the findings of this study are available from the corresponding author upon reasonable request. Scripts that were used for data analyses can be found at https://gitlab.ub.uni-bielefeld.de/ae02/emotionregulation_epilepsy.
