# Supplementary material for: Neural correlates of emotion processing and regulation dissociate frontal and temporal lobe epilepsy
Source: Brain Commun. 2026 Jul 28;8(4):fcag270. doi: 10.1093/braincomms/fcag270 (PMC13421894; doi:10.1093/braincomms/fcag270)
Supplement: fcag270_Supplementary_Data [file fcag270_supplementary_data.docx]

**Supplementary material**

**Supplement 1**

Semiological, EEG and MRI data for TLE patients

*Supplementary Table 1*

*L = left, R = right, > = more than, >> = propagating to, LT = long-term EEG recording, S = spot EEG, extern = recorded at another epilepsy center, *no histpathology available unless stated otherwise, **Please note that in none of the patients subsequently studied by invasive EEG did this result in a revision of the initial diagnosis.*

|  |  |  |  |  |  |
| --- | --- | --- | --- | --- | --- |
| **Localizing and lateralizing semiological information** | **EEG type (spot / long-term)** | **Interictal epileptiform EEG discharges** | **Ictal EEG onset** | **Epileptogenic MRI lesion*** | **Subsequent invasive EEG- monitoring**** |
| **Left TLE** |  |  |  |  |  |
| Aura (somatosensible, R side of the body), psychomotor | LT | L and R temporal | R temporal | L hippocampal sclerosis | Yes, at another center (extern). Seizure onset L > R hippocampus (36:1) |
| Abdominal aura, déjà vu, psychomotor | LT | L and R temporal | L temporal | None | No |
| Déjà vu, epigastric aura, aphasic | LT | L temporal | None | L temporal encephalocele | No |
| Dyscognitive, déjà-vu, fear aura | LT | L temporal | None | None | No |
| Epigastric aura, psychomotor, head version to the right | LT | L and R temporal | L temporal | None | No |
| Psychomotor, dystonic R arm | LT | L temporal | None | L hippocampal sclerosis | No |
| Psychomotor, dystonic R arm | LT | L temporal | None | None | No |
| Epigastric aura, psychomotor | S | L temporal | Not lateralizing or localizing | L hippocampal sclerosis | No |
| Psychomotor with postictal aphasia | LT | L temporal | L temporal | L temporal encephalocele | No |
| Psychomotor | LT | L temporal | L centro-parieto-temporal | L temporal large encephalocele | Yes, seizure onset near the L temporal encephalocele. |
| Psychomotor | LT | L fronto-temporal | None | L hippocampal sclerosis | Yes, seizure onset L medial temporal lobe. |
| Psychomotor, version to the R | no (extern LT) | L temporal | L temporal | None | No |
| Aphasic, psychomotor | LT | L temporal | None | L temporal tumor | No |
| Nonspecific | LT | L temporal | None | L hippocampal sclerosis | No |
| Psychomotor, aphasic (language fMRI: left-sided dominace), clonic movements right arm | LT | None | None | L temporal encephalocele | No |
| Déjà-vu | LT | L temporal | L temporal | Transient left hippocampal signal increase | No |
| Aphasic | LT | L temporal | None | L hippocampal sclerosis | No |
| Autonomic (pilomotor), psychomotor | LT | L > R temporal (5:1) | None | None | No |
| Psychomotor | LT | L temporal | None | L hippocampal sclerosis | No |
| Psychomotor | S (extern LT) | L temporal | None | L amygdalar enlargement | No |
| Psychomotor, post-ictal aphasia | No (extern LT) | None | None | L temporal lesion (biopsy: multinodular and vacuolating neuronal tumor, L temporal) | No |
| **Right TLE** |  |  |  |  |  |
| Vegetative aura, psychomotor (no postictal aphasia) | S | None | None | Amygdalar lesion, R > L side | No |
| Psychomotor | LT | R temporal | None | R amygdalar lesion | No |
| Psychomotor | LT | R temporal | R fronto-temporal | None | Yes, seizure onset R temporal >R frontal (8:1) |
| Dyscognitive (acustic hallucinations); no aphasia | LT | R temporal | R temporal | None | No |
| Psychomotor with ictal speech, orientation problems | LT | None | None | None | No |
| Psychomotor, déjà vu | LT | R temporal (82%) > L temporal (18%) | R temporal | R temporal tumor | No |
| Autonomic (epigastric or pilomotor aura) | LT | None | None | R hippocampal sclerosis | No |
| Psychomotor, epigastric, no post-ictal aphasia | S | None | None | R hippocampal sclerosis | No |
| Epigastric, psychomotor with nose-rubbing | LT | R temporal | None | R amygdalar enlargement | No |
| Psychomotor | LT | None | R fronto-temporal | R hippocampal sclerosis | No |
| Autonomic, disorientation | LT | R & L temporal | R temporal | None | No |
| Epigastric aura, psychomotor | LT | R temporal | R temporal | R hippocampal sclerosis | No |
| Déjà vu | LT | R temporo-occipital | None | None | No |
| Déjà vu, psychomotor | LT | None | R temporal | None | No |
| Psychomotor | No (extern LT) | None | R temporal | None | No |
| Epigastric, depersonalisation | LT | R temporal > L temporal | None | Mild signal increase R amygdala | No |
| Psychomotor, pilomotor | No (extern LT) | R temporal | R temporal | None | No |
| Psychomotor | No (extern LT) | R temporal | R temporal | R transient amygdala volume increase | No |
| **FLE** |  |  |  |  |  |
| Tonic seizures during sleep | LT | L frontal & fronto-central | None | None | No |
| Aphasic and psychomotor | LT | L & R frontal & fronto-temporal, L temporal | None | Bilateral frontal subependymal heterotopias | No |
| Hypermotor, psychomotor | LT | L & R frontal | None | L fronto-lateral and fronto-parieto-mesial low-grade focal cortical dysplasia | No |
| Tonic-clonic of R leg | No (extern S) | None | None | L fronto-parietal low-grade glioma | No |
| Head version to the R, psychomotor, absences | LT | L frontal and generalized | None | None | No |
| Hypermotor, tonic-clonic | LT | L & R frontal | R frontal | None | Yes, extern. Frontal seizure onset, No information on R or L available |
| Head version to the R, psychomotor | LT | L frontal | Generalized (during status epilepticus) | None | No |
| Clonic R face, march to R hand | LT | None | None | L frontal subependymal heterotopia | No |
| Absence-like | LT | L fronto-central | L fronto-central >> L hemisphere >> R hemisphere | None | No |
| Tonic-clonic | LT | L & R frontal | Generalized with frontal maximum | None | No |
| Vegetative, postictal motor aphasia | LT | L posterior temporal, L frontal sharp transient | None | L fronto-basal cavernoma, scar medial orbital gyrus (Broca; language fMRI: left-sided dominance) | No |
| Hypermotor, vegetative, psychomotor | LT | None | None | None | No |
| Tonic R | No (extern LT) | L frontal | L frontal | L frontal focal cortical dysplasia | No |
| Vegetative | LT | None | L frontal | None | No |
| Tonic-clonic with postictal L paresis | S | R fronto-centro-parietal | None | None | No |
| Dyscognitive, head version to the R | LT | R fronto-central, generalized | None | None | No |
| Version to the R, body rocking | No (extern LT) | L & R fronto-central | L & R fronto-central | None | No |
| Somatosenory R side of body | LT | L fronto-temporal | None | L frontal heterotopia | No |

**Supplement 2**

*
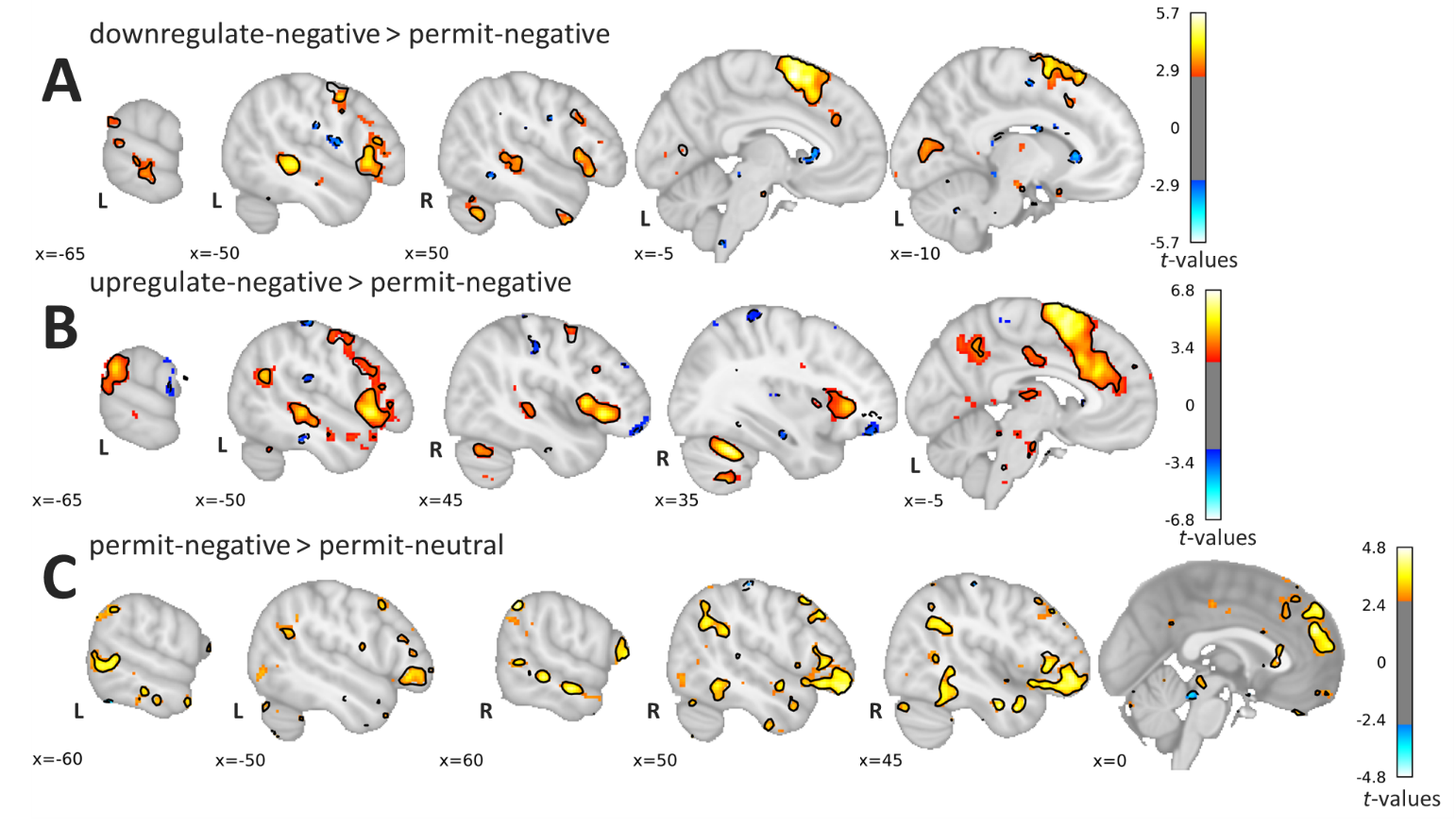
*

*Supplementary Fig 1*. Left temporal lobe epilepsy (lTLE) patients’ activation maps for downregulate-negative > permit-negative (panel A), upregulate-negative > permit-negative (panel B) and permit-negative > permit-neutral (panel C). Activation maps presented here are shown only for descriptive purposes to enable a descriptive comparison between the whole lTLE group (*n* = 21) and a subsample (*n* = 19), excluding two participants with seizures shortly before the functional magnetic resonance imaging task (black contours; see the methods section). To illustrate larger patterns, activation maps are shown at *p_uncorr_* < .005. Visual inspection shows that activation maps do not differ substantially whether or not these patients are included. Activation maps show within-group *t*-contrasts. L = left, R = right.

**Supplement 3**


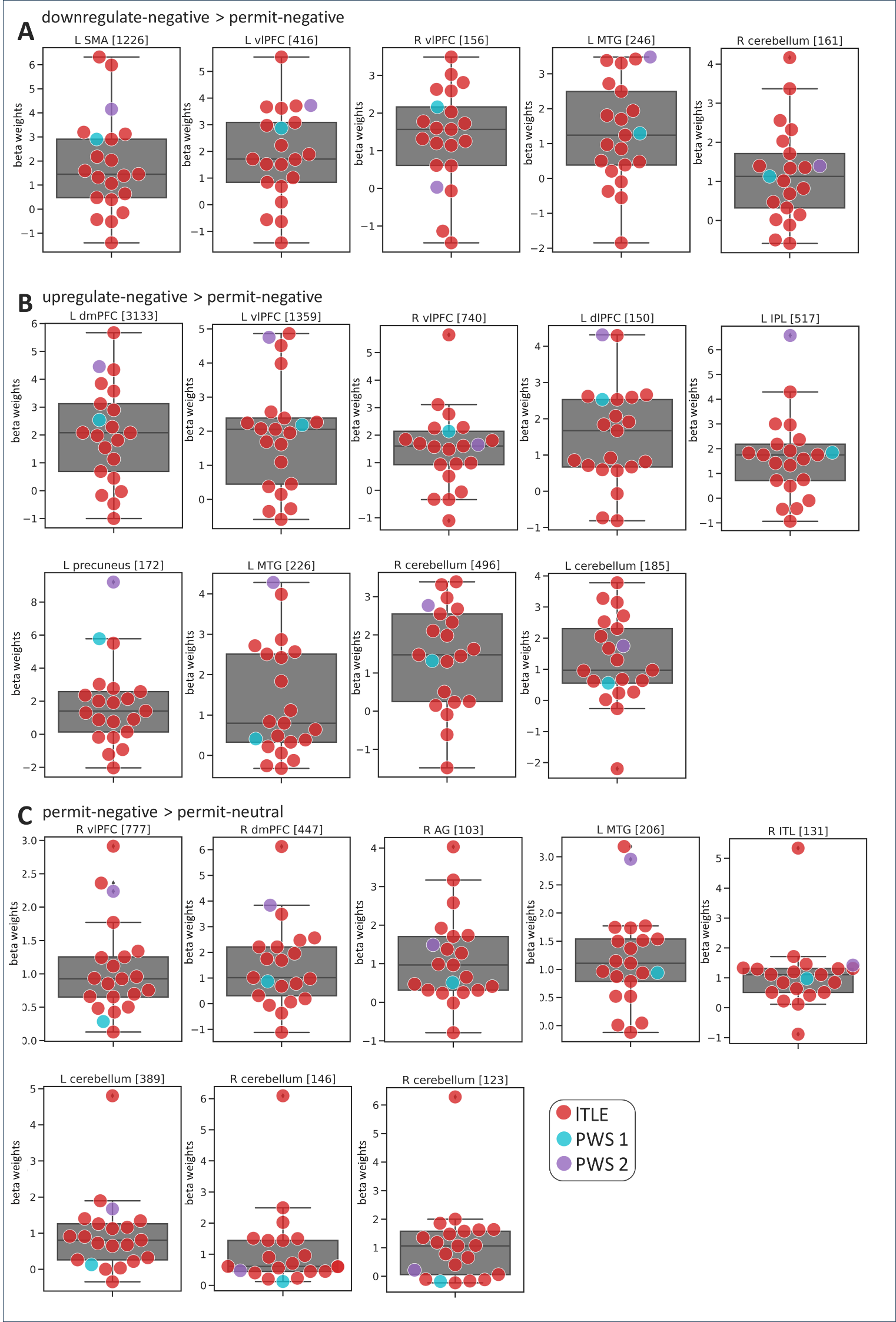


*Supplementary Fig. 2*. Boxplots display activations of left temporal lobe epilepsy (lTLE; *n* = 21) patients for the within-group *t*-contrasts downregulate-negative > permit-negative (panel A), upregulate-negative > permit-negative (panel B) and permit-negative > permit-neutral (panel C). The number in brackets denotes the cluster size in voxels. Dots represent individual participants. The y-axis shows beta weights. Boxplots show average cluster activity and are presented only for descriptive purposes, i.e. to show that the two participants with seizures (PWS) shortly before the functional MRI task (see the methods section) did not systematically differ from the rest of the group. Test statistics for condition comparisons within the whole lTLE group are based on SPM12’s second-level full factorial model (www.fil.ion.ucl.ac.uk/spm; see methods section) and are detailed in Supplementary Table 2. L = left, R = right; SMA = supplementary motor area, PFC = prefrontal cortex, vl = ventrolateral, dl = dorsolateral, dm = dorsomedial, MTG = middle temporal gyrus, IPL = inferior parietal lobe, AG = angular gyrus, ITL = inferior temporal lobe.

**Supplement 4**


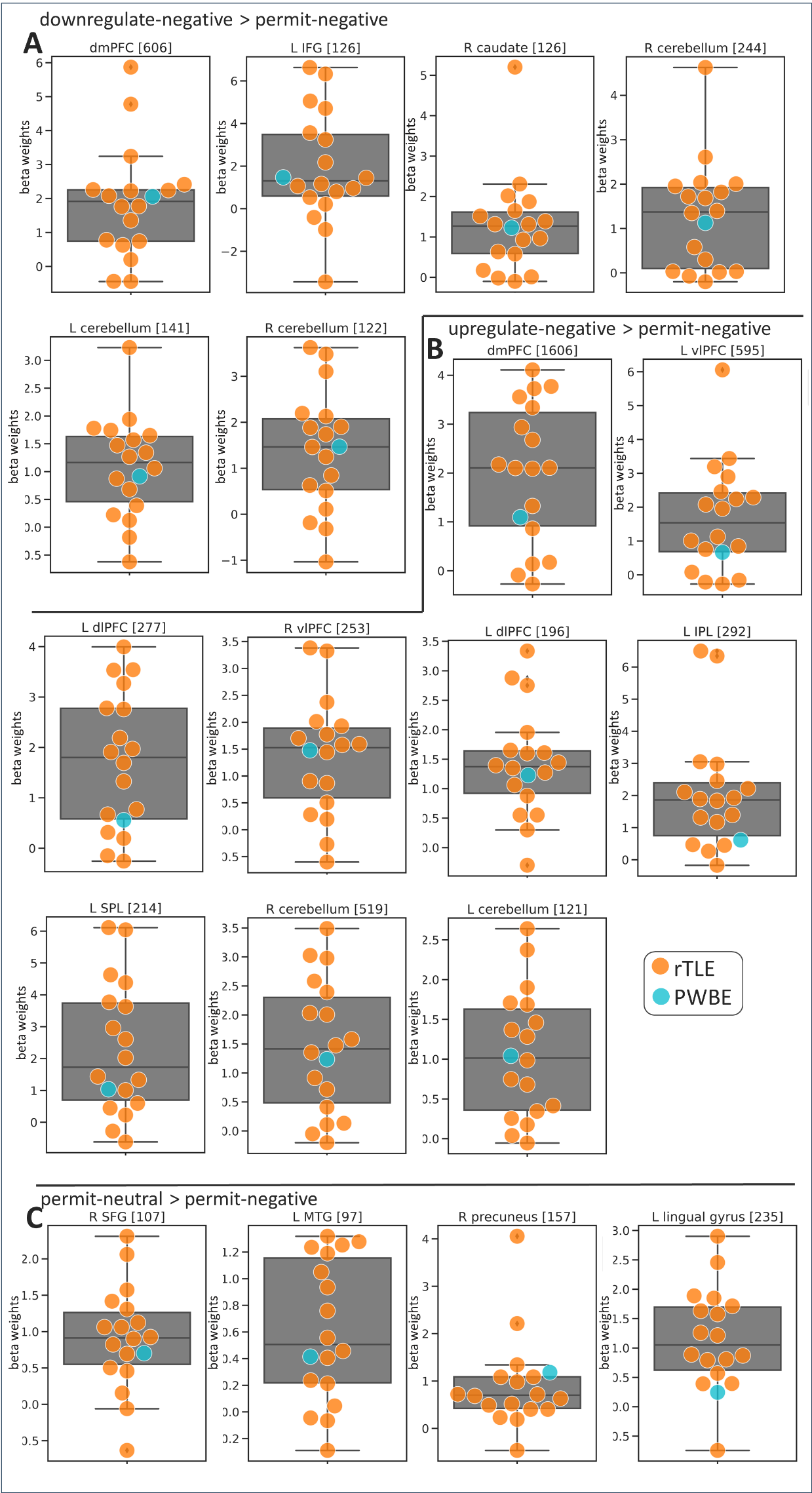


*Supplementary Fig. 3*. Boxplots display activations of right temporal lobe epilepsy (rTLE, *n* = 18) patients for the within-group *t*-contrasts downregulate-negative > permit-negative (panel A), upregulate-negative > permit-negative (panel B) and permit-neutral > permit-negative (panel C). The number in brackets denotes the cluster size in voxels. Dots represent individual participants. The y-axis shows beta weights. Boxplots show average cluster activity and are presented only for descriptive purposes, i.e. to show that one participant who was blind on the left eye (PWBE; see the methods section) did not systematically differ from the rest of the group. Test statistics for condition comparisons within the whole rTLE group are based on SPM12’s second-level full factorial model (www.fil.ion.ucl.ac.uk/spm; see methods section) and are detailed in Supplementary Table 2. L = Left, R = Right; PFC = prefrontal cortex, vl = ventrolateral, dl = dorsolateral, dm = dorsomedial, IFG = inferior frontal gyrus, SPL = superior parietal lobe, IPL = inferior parietal lobe, SFG = superior frontal gyrus, MTG = middle temporal gyrus.

**Supplement 5**

**
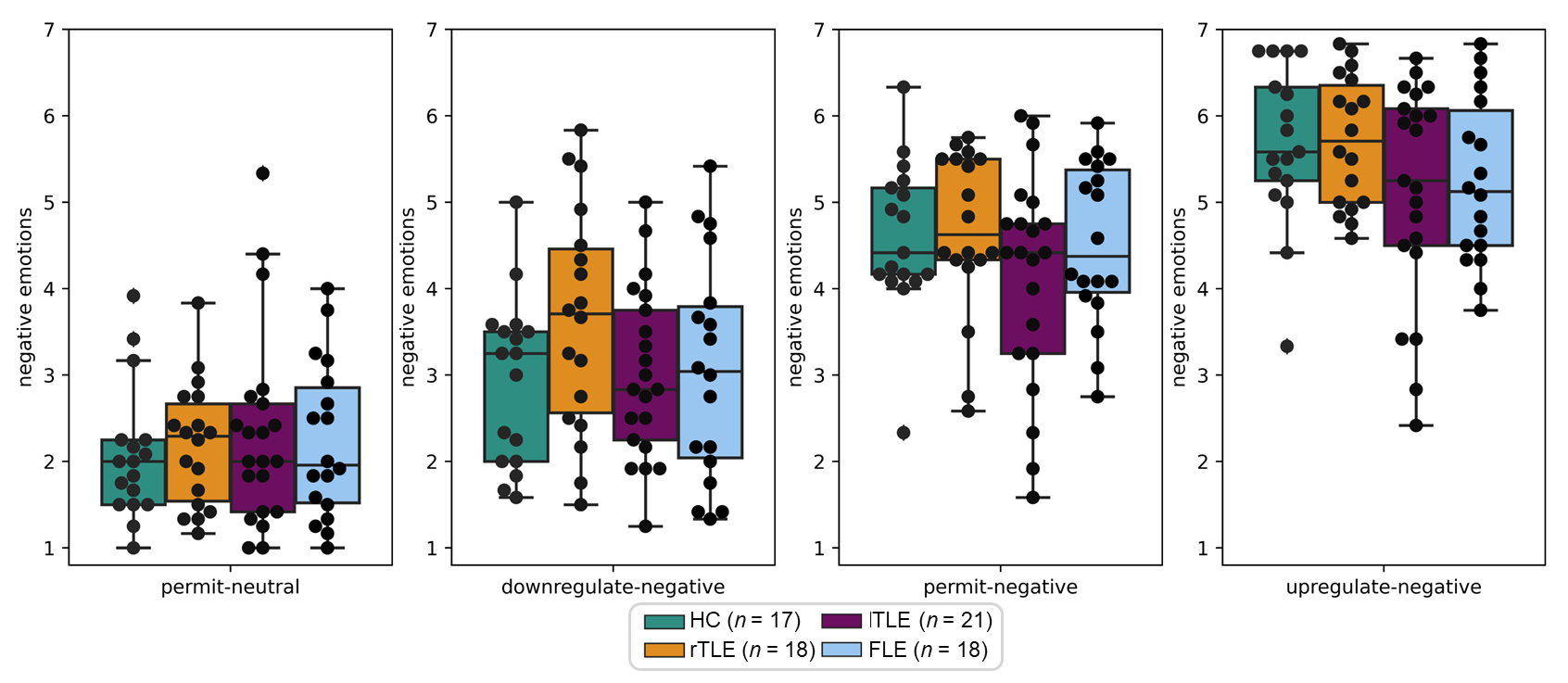
***Supplementary Fig. 4*. Self-reported negative affect on a seven-point Likert-scale after regulating or permitting emotions towards negative or neutral scenes. Black dots represent individual participants. Analysis of variance revealed that across groups, self-reported negative affect differed as expected by task (*F*(3,219) = 189.148, all *p_Bonferroni_* < .001). Groups did not differ overall in their ratings nor was there an interaction between group and task condition (all *p_uncorr_* > .10). HC = healthy controls, rTLE = right temporal lobe epilepsy, lTLE = left temporal lobe epilepsy, FLE = frontal lobe epilepsy.

**Supplement 6**

Whole Brain Results of Within-Group Contrast

*Supplementary Table 2*

*Activated clusters during downregulating, upregulating and permitting emotions towards negative or neutral picture for controls, left and right temporal lobe epilepsy (TLE) and frontal lobe epilepsy (FLE) patients. L = left, R = right, IFG = inferior frontal gyrus, n.a. = not assigned.*

| Side | Activation Peak | x | y | z | z score | Volume (voxels) | pFWE (cluster) |
| --- | --- | --- | --- | --- | --- | --- | --- |
| **Healthy Controls** | | | | | | | |
| **downregulate > permit** | | | | | | | |
| L | superior frontal gyrus | -10 | 6 | 70 | 5.24 | 1400 | < .001 |
| R | " | 12 | 10 | 68 | 5.20 |  |  |
| R | paracingulate gyrus | 4 | 24 | 42 | 4.79 |  |  |
| L | orbitofrontal cortex | -36 | 32 | -4 | 4.18 | 397 | < .001 |
| L | " | -42 | 38 | -4 | 4.17 |  |  |
| L | anterior insula | -42 | 16 | -4 | 3.91 |  |  |
| R | IFG, triangular part | 54 | 30 | -2 | 5.19 | 337 | < .001 |
| R | IFG, opercular part | 50 | 16 | 2 | 4.29 |  |  |
| R | orbitofrontal cortex | 40 | 32 | -12 | 3.89 |  |  |
| R | cerebellum | 32 | -62 | -32 | 4.31 | 332 | < .001 |
| R | " | 50 | -58 | -34 | 4.19 |  |  |
| R | " | 18 | -66 | -30 | 3.71 |  |  |
| L | cerebellum | -24 | -64 | -30 | 4.23 | 234 | < .001 |
| L | " | -28 | -56 | -32 | 4.05 |  |  |
| L | " | -38 | -62 | -32 | 3.88 |  |  |
| **upregulate > permit** | | | | | | | |
| L | anterior cingulate cortex | -4 | 26 | 30 | 6.80 | 4595 | < .001 |
| L | superior frontal gyrus | -12 | 4 | 70 | 6.37 |  |  |
| R | paracingulate gyrus | 2 | 20 | 42 | 5.64 |  |  |
| L | anterior insula | -38 | 12 | -6 | 5.47 | 2252 | < .001 |
| L | orbitofrontal cortex | -40 | 28 | -2 | 5.44 |  |  |
| L | IFG, opercular part | -44 | 14 | 0 | 5.35 |  |  |
| R | IFG, opercular part | 50 | 18 | 2 | 5.20 | 1144 | < .001 |
| R | anterior insula | 30 | 20 | -10 | 4.69 |  |  |
| R | IFG, triangular part | 52 | 24 | -4 | 4.50 |  |  |
| L | angular gyrus | -44 | -72 | 28 | 4.84 | 638 | < .001 |
| L | " | -50 | -60 | 26 | 4.70 |  |  |
| L | " | -42 | -52 | 18 | 4.02 |  |  |
| L | precuneus | -6 | -54 | 38 | 4.01 | 122 | .027 |
| L | pallidum | -10 | 6 | 0 | 4.80 | 628 | < .001 |
| L | thalamus | -10 | -2 | 8 | 4.72 |  |  |
| L | pallidum | -18 | 2 | -6 | 4.58 |  |  |
| R | putamen | 22 | 6 | 10 | 4.25 | 110 | .042 |
| R | cerebellum | 42 | -60 | -28 | 5.38 | 839 | < .001 |
| R | " | 48 | -66 | -30 | 5.36 |  |  |
| R | " | 34 | -50 | -32 | 5.16 |  |  |
|  | brainstem | 0 | -36 | -48 | 4.72 | 196 | .002 |
| R | n.a. | 6 | -50 | -42 | 4.46 |  |  |
| R | " | 6 | -42 | -44 | 4.17 |  |  |
| L | cerebellum | -40 | -52 | -30 | 4.55 | 131 | .019 |
| L | " | -32 | -50 | -32 | 4.37 |  |  |
| L | " | -50 | -56 | -32 | 3.43 |  |  |
| **permit > neutral** | | | | | | | |
| R | middle frontal gyrus | 48 | 26 | 30 | 3.88 | 150 | .010 |
| R | " | 54 | 32 | 28 | 3.69 |  |  |
| R | " | 50 | 24 | 44 | 3.58 |  |  |
| L | cerebellum | -16 | -74 | -30 | 4.74 | 129 | .021 |
| **neutral > permit** | | | | | | | |
| L | precuneus | -16 | -56 | 22 | 4.90 | 367 | < .001 |
| L | retrosplenial cortex | -14 | -46 | 8 | 4.57 |  |  |
| L | precuneus | -6 | -60 | 16 | 4.17 |  |  |
| R | precuneus | 14 | -56 | 22 | 4.83 | 239 | .001 |
| R | " | 20 | -52 | 26 | 4.72 |  |  |
| R | " | 18 | -50 | 18 | 4.31 |  |  |
| L | temporo-occipital fusiform cortex | -32 | -48 | -4 | 4.52 | 204 | .002 |
| L | lingual gyrus | -30 | -40 | -8 | 4.01 |  |  |
| L | parahippocampal gyrus | -24 | -32 | -18 | 3.77 |  |  |
| R | parahippocampal gyrus | 26 | -34 | -12 | 4.33 | 148 | .010 |
| R | lingual gyrus | 26 | -52 | -6 | 3.57 |  |  |
| R | " | 32 | -44 | -6 | 3.51 |  |  |
| **Left TLE** | | | | | | | |
| **downregulate > permit** | | | | | | | |
| L | supplementary motor area | -4 | 6 | 60 | 5.46 | 1226 | < .001 |
| L | " | -2 | 8 | 68 | 5.31 |  |  |
| L | " | -2 | 16 | 58 | 5.30 |  |  |
| L | orbitofrontal cortex | -44 | 18 | 4 | 4.82 | 416 | < .001 |
| L | IFG, triangular part | -54 | 26 | -6 | 3.94 |  |  |
| L | " | -50 | 28 | 14 | 3.59 |  |  |
| R | orbitofrontal cortex | 46 | 26 | -6 | 4.48 | 156 | .009 |
| R | IFG, opercular part | 50 | 18 | 4 | 3.86 |  |  |
| R | orbitofrontal cortex | 54 | 26 | -8 | 3.50 |  |  |
| L | middle temporal gyrus | -48 | -32 | -2 | 4.92 | 246 | .001 |
| L | " | -60 | -34 | 0 | 4.08 |  |  |
| L | superior temporal gyrus | -62 | -24 | 0 | 3.47 |  |  |
| R | cerebellum | 36 | -54 | -28 | 4.51 | 161 | .008 |
| **upregulate > permit** | | | | | | | |
| L | superior frontal gyrus | -12 | 4 | 74 | 6.36 | 3133 | < .001 |
| L | supplementary motor area | -8 | 8 | 62 | 6.15 |  |  |
| L | " | -2 | 0 | 70 | 5.90 |  |  |
| L | IFG, triangular part | -44 | 18 | 2 | 5.82 | 1359 | < .001 |
| L | IFG, opercular part | -52 | 16 | -2 | 5.62 |  |  |
| L | orbitofrontal cortex | -34 | 28 | -4 | 4.71 |  |  |
| R | orbitofrontal cortex | 46 | 24 | -4 | 5.47 | 740 | < .001 |
| R | IFG, opercular part | 46 | 12 | 4 | 5.24 |  |  |
| R | insula | 32 | 28 | 4 | 4.98 |  |  |
| L | middle frontal gyrus | -34 | 2 | 60 | 4.54 | 150 | .012 |
| L | precentral gyrus | -54 | 2 | 48 | 3.90 |  |  |
| L | supplementary motor area | -42 | -2 | 62 | 3.68 |  |  |
| L | supramarginal gyrus | -64 | -46 | 30 | 4.69 | 517 | < .001 |
| L | angular gyrus | -54 | -58 | 24 | 4.61 |  |  |
| L | " | -40 | -54 | 24 | 4.23 |  |  |
| L | precuneus | -6 | -56 | 44 | 4.14 | 172 | .006 |
| L | " | -6 | -66 | 44 | 3.33 |  |  |
| L | middle temporal gyrus | -50 | -30 | -2 | 4.68 | 226 | .001 |
| L | " | -52 | -22 | -8 | 4.61 |  |  |
| R | cerebellum | 38 | -54 | -30 | 6.06 | 496 | < .001 |
| R | " | 44 | -64 | -28 | 4.11 |  |  |
| R | " | 14 | -66 | -28 | 3.73 |  |  |
| L | cerebellum | -34 | -56 | -30 | 5.26 | 185 | .004 |
| L | " | -34 | -64 | -24 | 3.99 |  |  |
| L | " | -44 | -56 | -30 | 3.90 |  |  |
| **permit > neutral** | | | | | | | |
| R | IFG, triangular part | 58 | 30 | 8 | 4.32 | 777 | < .001 |
| R | orbitofrontal cortex | 36 | 30 | -16 | 4.27 |  |  |
| R | frontal pole | 46 | 44 | -6 | 4.23 |  |  |
| R | paracingulate gyrus | 0 | 50 | 28 | 4.45 | 447 | < .001 |
| R | superior frontal gyrus | 2 | 50 | 40 | 4.07 |  |  |
| R | anterior cingulate cortex | 8 | 48 | 12 | 3.88 |  |  |
| L | middle temporal gyrus | -56 | -52 | 0 | 4.44 | 206 | .002 |
| L | inferior lateral occipital cortex | -56 | -62 | 4 | 3.90 |  |  |
| L | middle temporal gyrus | -68 | -48 | 2 | 3.60 |  |  |
| R | temporooccipital fusiform gyrus | 40 | -46 | -16 | 4.13 | 131 | .023 |
| R | inferior temporal gyrus | 48 | -46 | -12 | 3.77 |  |  |
| R | temporooccipital fusiform gyrus | 44 | -48 | -24 | 3.70 |  |  |
| L | cerebellum | -26 | -84 | -36 | 4.65 | 389 | < .001 |
| L | " | -34 | -78 | -42 | 4.40 |  |  |
| L | " | -30 | -82 | -28 | 3.97 |  |  |
| R | cerebellum | 32 | -78 | -32 | 4.40 | 146 | .013 |
| R | inferior lateral occipital cortex | 40 | -78 | -24 | 4.22 |  |  |
| R | cerebellum | 26 | -72 | -32 | 3.93 |  |  |
| R | cerebellum | 22 | -76 | -24 | 4.10 | 123 | .030 |
| R | " | 4 | -86 | -26 | 3.81 |  |  |
| R | " | 16 | -78 | -30 | 3.72 |  |  |
| **Right TLE** | | | | | | | |
| **downregulate > permit** | | | | | | | |
| L | paracingulate gyrus | -4 | 18 | 48 | 4.39 | 606 | < .001 |
| R | superior frontal gyrus | 6 | 10 | 60 | 4.29 |  |  |
| L | paracingulate gyrus | -4 | 10 | 52 | 4.21 |  |  |
| L | IFG, opercular part | -56 | 18 | 28 | 3.94 | 126 | .025 |
| L | IFG, triangular part | -50 | 26 | 24 | 3.50 |  |  |
| R | caudate nucleus | 10 | 14 | 6 | 4.19 | 126 | .025 |
| R | " | 16 | 12 | 12 | 3.82 |  |  |
| R | cerebellum | 36 | -60 | -26 | 4.60 | 244 | .001 |
| R | " | 32 | -52 | -32 | 4.60 |  |  |
| R | " | 50 | -56 | -38 | 3.72 |  |  |
| L | cerebellum | -38 | -56 | -32 | 4.16 | 141 | .014 |
| L | " | -48 | -56 | -34 | 3.87 |  |  |
| L | " | -44 | -64 | -28 | 3.83 |  |  |
| R | cerebellum | 38 | -80 | -28 | 4.22 | 122 | .028 |
| R | " | 30 | -80 | -26 | 3.99 |  |  |
| R | " | 18 | -84 | -24 | 3.64 |  |  |
| **upregulate > permit** | | | | | | | |
| L | paracingulate gyrus | -2 | 16 | 48 | 5.19 | 1606 | < .001 |
| L | anterior cingulate cortex | -4 | 18 | 34 | 4.93 |  |  |
| R | superior frontal gyrus | 10 | 6 | 72 | 4.42 |  |  |
| L | insula | -38 | 12 | 2 | 4.42 | 595 | < .001 |
| L | IFG, triangular part | -32 | 26 | 10 | 4.36 |  |  |
| L | " | -44 | 20 | 0 | 4.26 |  |  |
| L | superior frontal gyrus | -28 | 4 | 70 | 4.61 | 277 | < .001 |
| L | middle frontal gyrus | -38 | 2 | 60 | 4.15 |  |  |
| L | superior frontal gyrus | -18 | 4 | 64 | 3.92 |  |  |
| R | IFG, triangular part | 44 | 22 | 0 | 4.44 | 253 | < .001 |
| R | " | 54 | 30 | 2 | 3.99 |  |  |
| R | insula | 38 | 14 | 6 | 3.94 |  |  |
| L | superior frontal gyrus | -24 | 22 | 50 | 4.45 | 196 | .002 |
| L | " | -22 | 22 | 64 | 4.20 |  |  |
| L | middle frontal gyrus | -26 | 14 | 54 | 4.13 |  |  |
| L | angular gyrus | -48 | -78 | 34 | 4.85 | 292 | < .001 |
| L | " | -52 | -72 | 24 | 4.13 |  |  |
| L | superior parietal lobe | -4 | -62 | 62 | 4.84 | 214 | .001 |
| L | " | -2 | -52 | 70 | 3.49 |  |  |
| R | cerebellum | 36 | -60 | -26 | 5.77 | 519 | < .001 |
| R | " | 32 |  | -32 | 5.47 |  |  |
| R | " | 48 | -48 | -36 | 3.79 |  |  |
| L | cerebellum | -34 | -48 | -32 | 4.58 | 121 | .030 |
| L | " | -48 | -54 | -34 | 3.89 |  |  |
| **neutral > permit** | | | | | | | |
| R | superior frontal gyrus | 20 | 20 | 56 | 4.29 | 107 | .050 |
| R | " | 22 | 28 | 56 | 3.97 |  |  |
| R | " | 24 | 34 | 50 | 3.69 |  |  |
| R | precuneus | 14 | -50 | 22 | 4.51 | 157 | .008 |
| R | " | 24 | -58 | 20 | 3.98 |  |  |
| R | " | 14 | -58 | 26 | 3.24 |  |  |
| L | parahippocampal gyrus | -32 | -40 | -8 | 5.34 | 235 | .001 |
| L | lingual gyrus | -26 | -44 | -4 | 5.21 |  |  |
| L | " | -22 | -58 | -10 | 3.88 |  |  |
| **FLE** | | | | | | | |
| **permit > neutral** | | | | | | | |
| L | superior parietal lobe | -34 | -70 | 58 | 4.54 | 147 | 0.012 |
| L | " | -16 | -74 | 62 | 4.17 |  |  |
| L | " | -26 | -72 | 60 | 3.92 |  |  |

**Supplement 7**
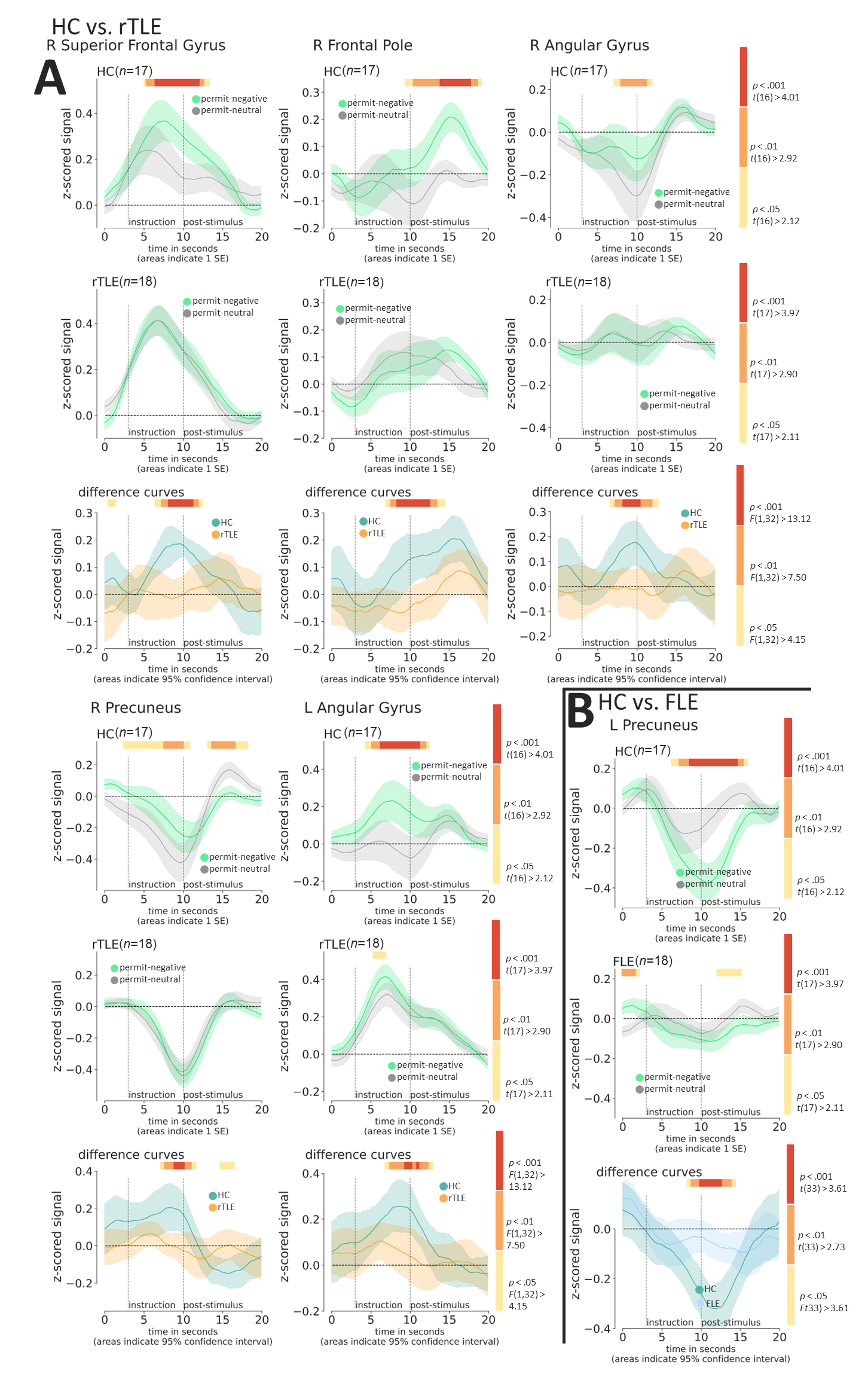


*Supplementary Fig. 5.* Time courses and difference curves for permit-negative versus permit-neutral, computed for regions, in which healthy controls (HC; *n* = 17) differed from right temporal lobe epilepsy (TLE; *n* = 18) patients (panel A) and from frontal lobe epilepsy (FLE; *n* = 18) patients (panel B). For within-group analyses, one-sample *t*-tests for differences between conditions were computed for each time-point. For between-group analyses two-sample *t*-tests, or ANCOVAs, were computed for each timepoint. *SE* = standard error of the mean. L = left, R = right.

**Supplement 8**
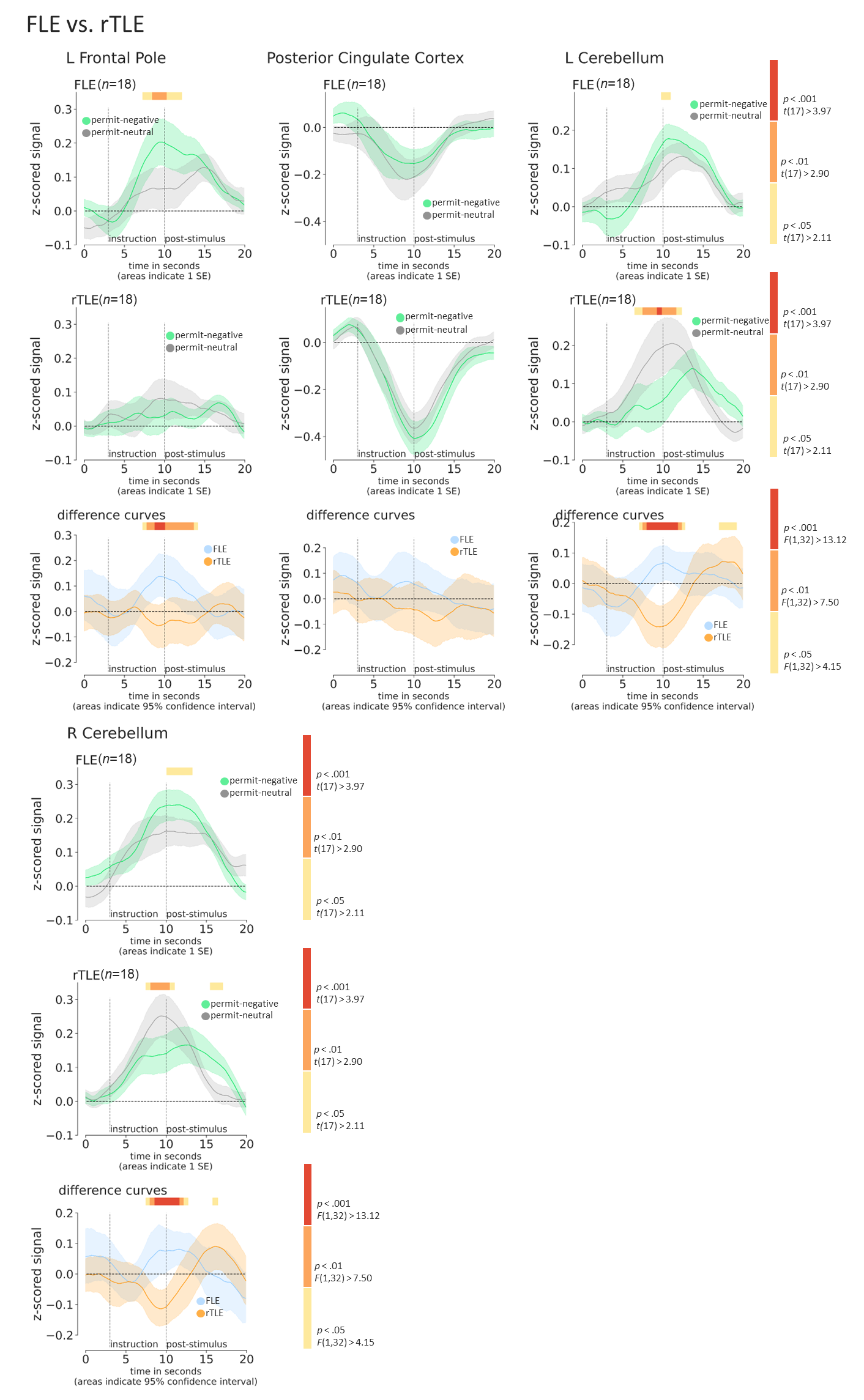


*Supplementary Fig. 6.* Time courses and difference curves for permit-negative versus permit-neutral, computed for regions, in which FLE (*n* = 18) patients differed from right TLE patients (*n* = 18). For within-group analyses, one-sample *t*-tests for differences between conditions were computed for each time-point. For between-group analyses ANCOVAs were computed for each timepoint. *SE* = standard error of the mean. L = left, R = right.

**Supplement 9**
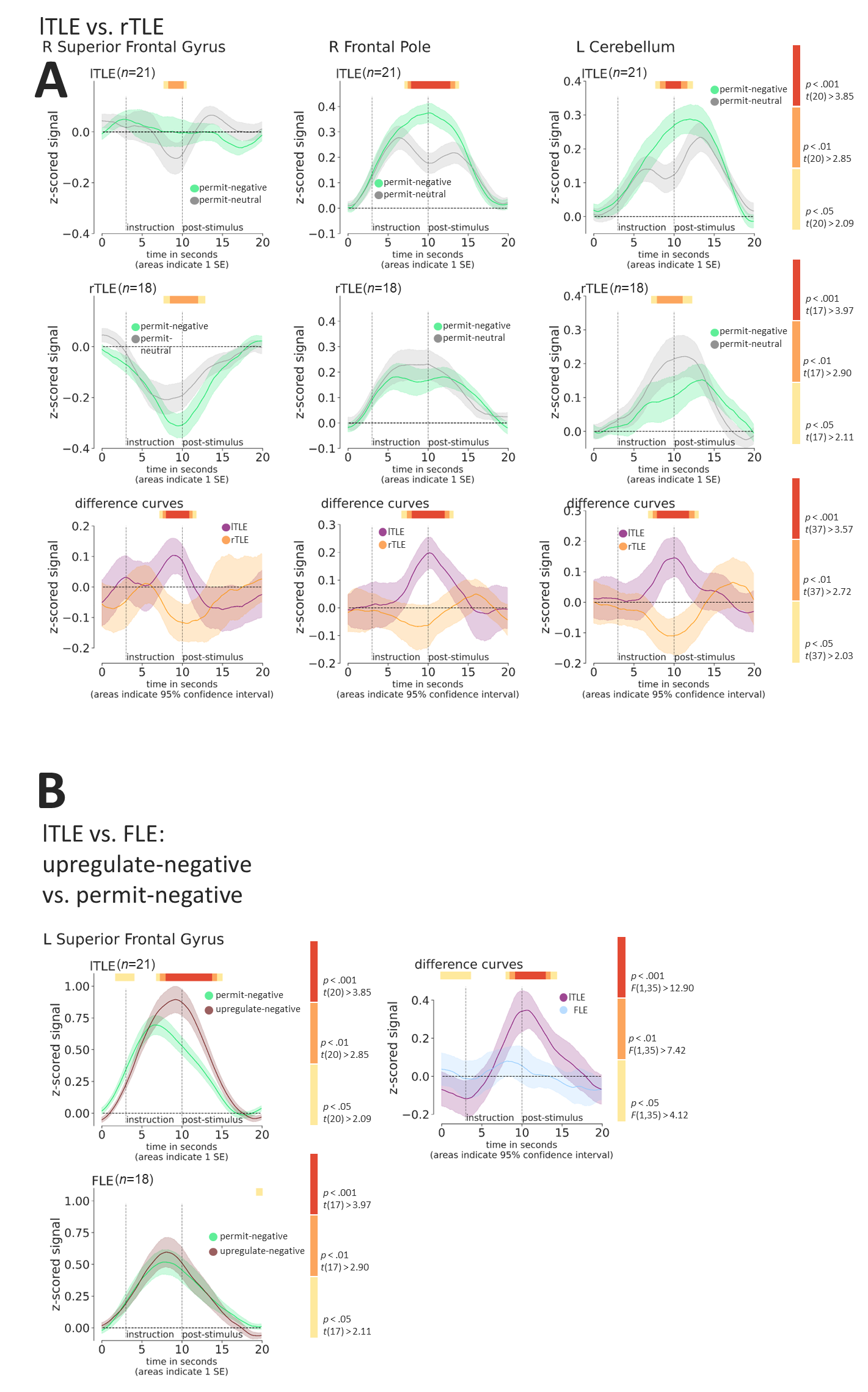


*Supplementary Fig. 7.* Panel A: Time courses and difference curves for permit-negative versus permit-neutral, computed for regions, in which left TLE patients (*n* = 21) differed from right TLE patients (*n* = 18). Panel B: Time courses and difference curves for upregulate-negative versus permit-negative, computed for the left superior frontal gyrus for left TLE and FLE patients (*n* = 18). For within-group analyses, one-sample *t*-tests for differences between conditions were computed for each time-point. For between-group analyses two-sample *t*-tests, or ANCOVAs, were computed for each timepoint. *SE* = standard error of the mean. L = left, R = right.

**Supplement 10**


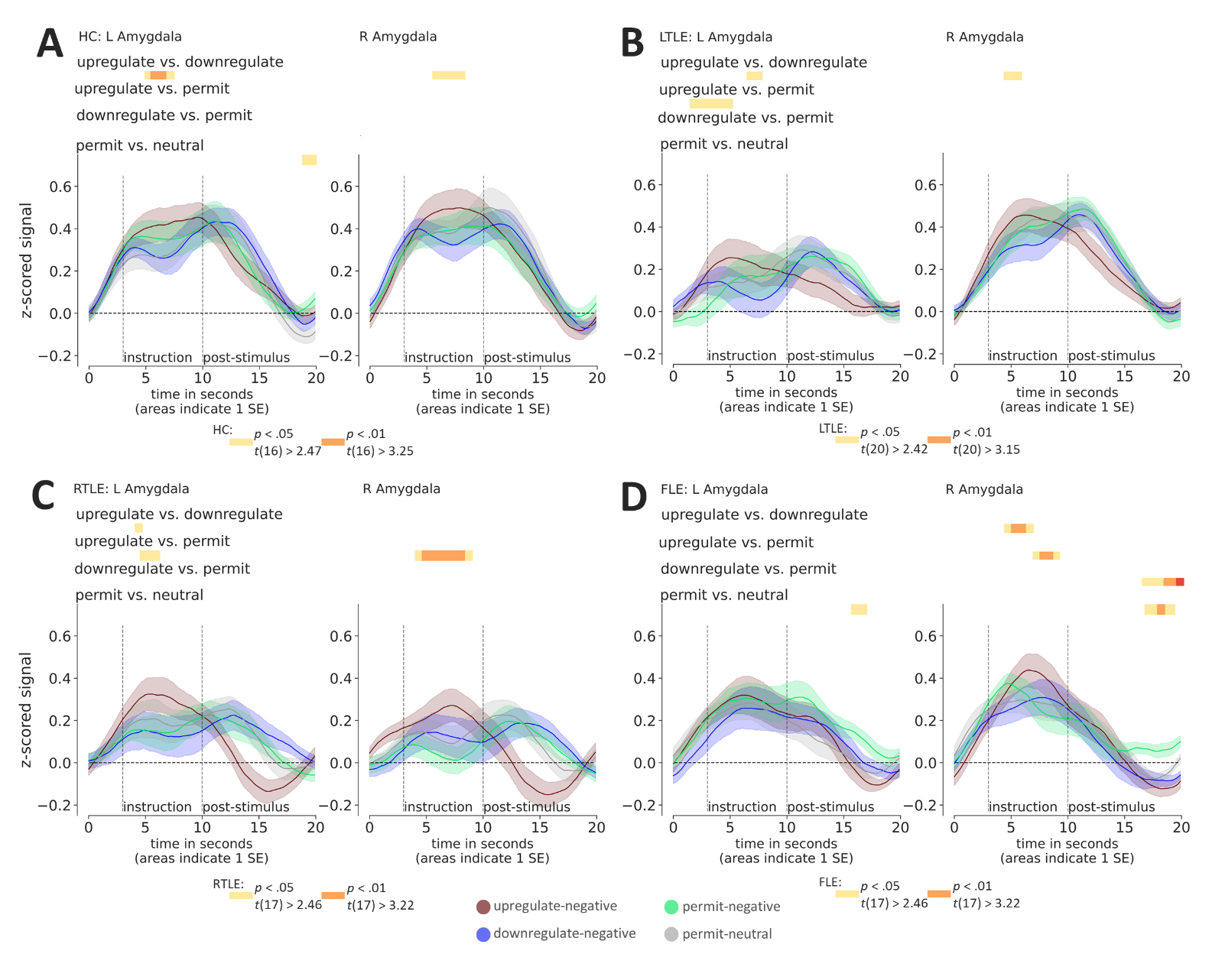


*Supplementary Fig. 8*. Time courses for healthy controls (HC; panel A; *n* = 17), left temporal lobe epilepsy (lTLE) patients (panel B; *n* = 21), right temporal lobe epilepsy (rTLE) patients (panel C; *n* = 18) and FLE patients (panel D; *n* = 18). One-sample t-tests for differences between conditions were computed for each time-point, separately for each group. Hypotheses were tested one-tailed, assuming that upregulate-negative > downregulate-negative, upregulate-negative > permit-negative, permit-negative > downregulate-negative and permit-negative > permit-neutral. Bonferroni-corrected *p*-values are reported. HC and FLE time courses are adapted from Benzait et al., 2023. *SE* = standard error of the mean. L = left, R = right.

**References**

1. Benzait A, Krenz V, Wegrzyn M, et al. Hemodynamic correlates of emotion regulation in frontal lobe epilepsy patients and healthy participants. *Hum Brain Mapp*. 2023;44(4):1456-1475. doi:10.1002/hbm.26133
